# Characterization of cosmetic sticks at Xiaohe Cemetery in early Bronze Age Xinjiang, China

**DOI:** 10.1038/srep18939

**Published:** 2016-01-28

**Authors:** Huijuan Mai, Yimin Yang, Idelisi Abuduresule, Wenying Li, Xingjun Hu, Changsui Wang

**Affiliations:** 1 Key Laboratory of Vertebrate Evolution and Human Origins of Chinese Academy of Sciences, Institute of Vertebrate Paleontology and Paleoanthropology, Chinese Academy of Sciences, Beijing 100044, People’s Republic of China; 2 Department of Archaeology and Anthropology, University of Chinese Academy of Sciences, Beijing 100049, People’s Republic of China; 3 Xinjiang Cultural Relics and Archaeology Institute, Ürümchi 830000, People’s Republic of China

## Abstract

Cosmetics have been studied for a long time in the society and culture research, and its consumption is regarded as a cultural symbol of human society. This paper focuses on the analysis of the red cosmetic sticks, found in Xiaohe Cemetery (1980–1450BC), Xinjiang, China. The structure of the red cosmetic sticks was disclosed by SR-μCT scanning (Synchrotron Radiation Micro-computed Tomography), while the chemical components were characterized by FTIR (Fourier Transform Infrared Spectroscopy), Raman Spectroscopy and Proteomics. The results suggested that the cosmetic sticks were made from the cattle heart and covered with a layer of hematite powders as the pigment. Given the numerous red painted relics in Xiaohe Cemetery, this kind of cosmetic sticks might be used as a primitive form of crayon for makeup and painting. The usage of cattle hearts as cosmetic sticks is firstly reported up to our knowledge, which not only reveals the varied utilizations of cattle in Xiaohe Cemetery but also shows the distinctive religious function. Furthermore, these red cosmetic sticks were usually buried with women, implying that the woman may be the painter and play a special role in religious activities.

Painting is one of the art forms used to express human thoughts. Among various types of painting, face painting, as an important part of cosmetic, always has special meaning. Painting or tattoo on human face could directly exhibit cultural connotations. Thus, cosmetic has a close relationship with human and develops with culture evolution. In the Upper Palaeolithic Age, the hematite was found around the buried human bones and was presumably related to painting, which might be a kind of cosmetic^1^. The goddess head (c. 5000 BP) with red-painted cheek and lip found in Niuheliang site in China showed that the cosmetic had been commonly practised in prehistoric times^2^. The functions of cosmetic were summarized as^3^: 1) aesthetic, the pursuit of beauty; 2) hygienic and therapeutic, for example, ancient people used particular cosmetic to protect their eyes or skin^4^; 3) religious functions, hunting camouflage or religion worship expression^5^,^6^, for instance, in Li Nationality, a minority in China, the face painting in a woman is considered as a symbol of frog worship^7^. Since cosmetic is a significant manifestation of human culture and attracts increasing attention, some researchers focus on the culture and social characteristics of cosmetic patterns and colours through the historic literatures and related relics, such as Zhou summarizes the feature of facial cosmetics, hair accessories, earrings and jewellery in historic periods in China^8^; Li presents the different materials, tools, raw materials, manufacture methods and working efficiency of Chinese traditional makeup^9^. Specially, the inorganic and organic components of the excavated cosmetics have been identified to get more information^3^,^4^,^10^,^11^,^12^,^13^,^14^,^15^,^16^,^17^,^18^,^19^,^20^,^21^. However, there was little study about the tools of cosmetic, mainly because the cosmetic tools were rarely found in excavation and sometimes it was difficult to confirm whether remains were cosmetic tools or not without detailed and further analysis. Studying cosmetic tools and figuring out their compositions can help understand the detailed process of makeup. Besides that, the production of the tools might reflect the exploitation of animal and plant resources, or other aspect of the contemporary society. For example, the analysis of ancient crayons from Cave Loncomán confirmed the use of animal source in the manufacture of the pastes^22^. Wang^23^ summarized the cosmetic sticks unearthed in Xinjiang of China, listed and compared the characteristics and made the gender study about the usage of these cosmetic sticks, but no scientific analysis was carried out on their production technology up to now. The further study about cosmetic tools would help people understand more about the prehistory society and culture in Xinjiang.

Xiaohe Cemetery (40°20′11″N, 88°40′20.3″E; c.1980–1450BC) is one of the most important Bronze Age sites in Xinjiang, China. It enjoyed a high reputation all around the world because it showed a mysterious and wonderful culture 3500 years ago. This site won the honour as one of the top 10 important archaeological discoveries of 2004 in China. This site is located in the Lop Nur, about 60 km south of Peacock River and 102 km west of ancient Loulan City (Fig. 1), and was comprehensively excavated from 2002 to 2004^24^,^25^. Due to the extremely dry and hot environment, a large number of organic relics were preserved well. As an important representative site of Xiaohe Culture, which prevailed in the central and eastern of Tarim Basin in Xinjiang about 4000 years ago, Xiaohe Cemetery revealed the unique cultural feature. This site was composed of five layers burials. The human bodies and funerary objects were placed in the wooden boat-shaped coffins wrapped by cattle hides. A huge wooden pillar, whose shape depended on the gender of the tomb occupier, stood in front of each coffin. Archaeologists believed that these pillars are a kind of reproduction worship^26^. Because of the important geographic location, time and cultural feature, research about the Xiaohe Cemetery remains would provide more information about the Eurasian east-west cultural exchange. For example, molecular analysis revealed that the residents in Xiaohe had the appearance of Europa, but more complicated genetic structure^27^. The utilization of plant resource in Xiaohe included ephedra, wheat, millet, love grass, etc^28^. The cow, the most commonly used of animal resource, was found that it was closer to the western Eurasia domesticated cattle^27^,^29^. In addition, the use of dairy products in Xiaohe, especially the kefir cheese, was of a great significance^30^.

In this site, face painting was common on mummies, especially the red lines on the forehead. Notably, most of female mummies were buried with a leather bag, a wooden phallus and wooden combs, as typically indicated by the occupier of tomb M13 (Fig. 2). These stuffs were usually near the waist of the mummy and the wooden phallus is a symbol of reproduction worship^31^. Red cosmetic sticks were found inside most of the leather bags and were presumably used to paint the face of mummy^24^,^25^,^32^.

Two red cosmetic sticks were selected from the leather bags found in Tomb M17 and M22, Which was found in layer 1 and layer 2 respectively, dated to 1650–1450 BC^25^, and labelled as stick 1 and stick 2 respectively (Fig. 3). Then, both sticks were analysed by various technologies: SR-μCT was used to disclose the internal structure; Raman spectrum was used to identify inorganic pigments; FTIR was used to evaluate the nature of organic materials and proteomics analysis was employed subsequently to identify the protein composition and their biological origin. Accordingly, the exploitation of natural resources in the Xiaohe Cemetery and the development of cosmetic tools in Xinjiang area would be discussed.

## Results

### Microscopy analysis

**Figure 3:**
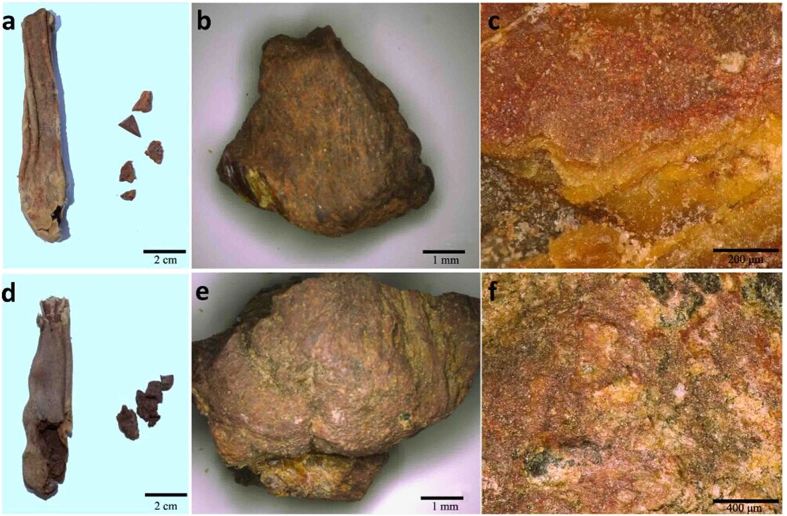
The leather bag and cosmetic sticks from tomb M17 (**a**) and tomb M22 (**d**); the analyzed sample: stick 1 (**b**) and stick 2 (**e**); the surface observed by microscope of stick 1 (**c**) and stick 2 (**f**).

Under optical microscope, there were some red particles on the stick surface (Fig. 3 c and f). Furthermore, the inner layer was yellow and translucent somewhere. These two obviously different coloured layers implied that they should be made of different materials.

**Figure 4:**
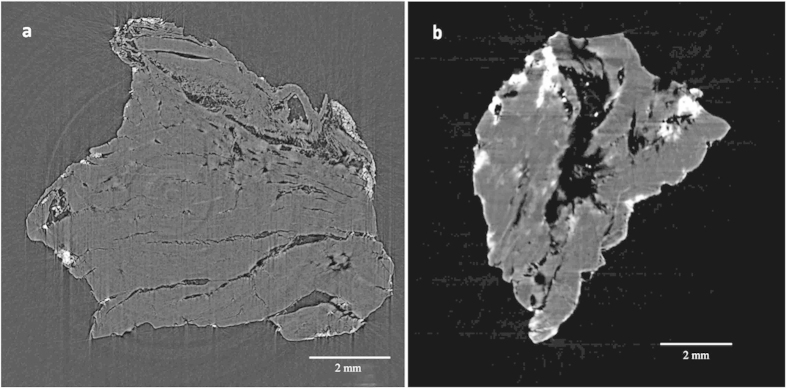
The CT slices of the cross section of stick 1 (**a**) and stick 2 (**b**).

SR-μCT scanning provided the crosss sections of both sticks (Fig. 4), which had the similar structure: the main part of the sticks was gray, but some brighter areas scattered on the surface, indicating two kinds of materials with different densities. In the CT slice, the grayscale standed for the object’s absorption of X-ray. Usually the heavy elements would have higher X-ray absorption and showed brighter in CT slices^33^. According to the grayscale, the main part of the sticks should be made of organic materials and some inorganic materials scatter on the surface.

### Pigment identification

**Figure 5:**
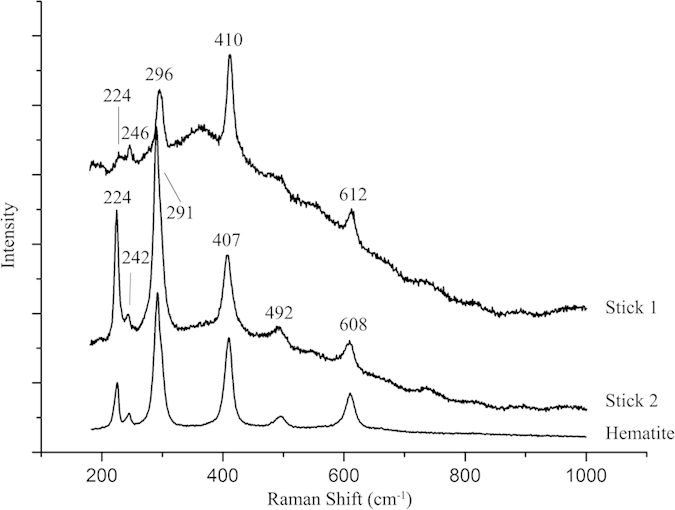
Raman Spectra of the hematite reference and the red particles on stick 1 and stick 2.

The Raman analysis was used to identify the red particles on the stick surface. The recognizable peaks of the red particles were showed in Fig. 5. The Raman spectra of two samples were compared with the Raman spectrum of hematite downloaded from the RRUFF database (Hematite R060190) and they had almost the same main peaks. The peaks at 224,246, 296, 410 and 612 cm^−1^ in the spectrum of stick1 and the peaks at 224, 242, 291,407, 492 and 608 cm^−1^ in the spectrum of stick2 were characteristic peaks of hematite (Fe_2_O_3_)^34^. The same characteristic peak would have small shifts in different spectra, which might be caused by complex reasons, such as the different instruments and parameters, influence of burial environment and tiny difference in the crystal structure. For example, Yang *et al.* pointed out that the longer the burial time, the weaker the main Raman peaks of chalcedony on the surface of stone axe might be^35^.

### Organic residue analysis

The FTIR was used to analyse the inner material (yellow and translucent). The spectra of both sticks were presented in Supplementary Materials Fig. S1. Compared with literature data^36^,^37^, the spectra reveal the presence of protein, whose characteristic signals was the amide group (-N(H)-C = O-). More specifically, the peak at 3290–3300 cm^−1^ was assigned to N-H stretching vibration region; around 1650 cm^−1^ to C = O stretching vibration region; around 1540 cm^−1^ to N-H bending vibration region, and 1400–1420 cm^−1^ to C-N stretching region. These four functional groups belonged to the same molecule, e.g. an amino acid. The results suggested that the organic materials of the sticks were mainly made of protein.

Thus, proteomics was carried out to identify the nature and species of the protein materials of the sticks. Each sample had been analysed twice to gain enough information about the protein composition. Except for disregarded human background proteins, such as the keratin, all the identified peptides were BLAST searched against the NCBInr database to check the species-specificity of the sequences. The identified proteins and the specific peptides were listed in Table 1, Supplementary Materials Table S1 and S2, respectively.

The identified proteins contained myosin, actin, muscle auxiliary protein, collagen and others. Myosin is the main constituent units of myofibrils, which has two light chains and two long chains, and is also the components of the cytoskeleton^38^. Actin is a structural protein of microfilaments, which is the main protein of the cytoskeleton, and is widespread in the various types of muscle tissue and cell; especially the alpha-actin plays an important role in the regulation of the muscle fibers^39^. Titin, desmin and myomesin also belong to cytoskeletal proteins of muscle^40^. Besides, the collagen is the major component of connective tissue and serum albumin is the most abundant protein in animal plasma. According to the dataset of identified proteins, it was obvious that the inner material of sticks contained the muscle tissue. Notably, the myosin-binding protein C was identified as the cardiac-type. In addition, the type VI collagen was found and certified to exist in the heart tissue in previous work^41^,^42^. Thus, the sticks were made of animal heart.

**Figure 6:**
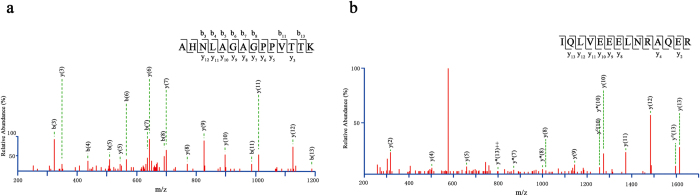
MS/MS spectra confidently identified two specific peptide sequences: (**a**) AHNLAGAGPPVTTK from stick 1 and (**b**) IQLVEEELNRAQER form stick 2.

According to the results of BLAST, the specific peptides equally present in cattle (*Bos taurus*), bison (*Bison bison bison*), yak (*Bos mutus*), zebu (*Bos indicus*), domestic yak (*Bos grunniens*), European bison (*Bison bonasus*) and water buffalo (*Bubalus bubalis*). The MS/MS spectra of bovine-specific peptides, such as AHNLAGAGPPVTTK from myosin-binding protein C, cardiac-type in stick1 and IQLVEEELNRAQER from tropomyosin 4-like in stick2 were shown in Fig. 6. Although the complete zooarchaeological data of Xiaohe Cemetery are not published, other species of bovine bones except of cattle have been not found^28^. Furthermore, some DNA analyses identified bovine remains in Xiaohe Cemetery as *Bos taurus*^27^,^29^. And the desert environment in Xiaohe is not suitable for yak to live. Thus, the cattle is the most possible interpretation for the origin of these specific peptides and the organic part of both sticks is from cattle heart.

## Discussion

The red cosmetic sticks analysed were made of cattle heart covered with hematite powders. Hematite was one of the most widely used red pigments with the development of human being. About 18000 years ago, the hematite powder was found around the bones at Zhoukoudian, Beijing^1^. It was a common phenomenon of using hematite to paint red on human skull and axe in Qijia Culture and Xindian Culture thousands of years ago^43^. During the historical period, besides widely used as a kind of pigment in painting, mural painting and coloured painting sculpture, hematite is also treated as a traditional mineral drug to cure disease in China^44^. However, it was firstly reported that the cattle heart was used as cosmetic stick up to our knowledge, and the myocardium contained a certain amount of fat and collagen^45^, which were traditional and natural adhesives so as to attach the pigments conveniently to paint. And these sticks were irregular, which might be residue after painting or caused by the dehydration of muscle. Besides as the cosmetic tool, the stick also might be a kind of early painting tool, like brush or crayon, supported by plenty of relics painted red in Xiaohe Cemetery. Up to now, the earliest unearthed brush made of bamboo and rabbit hair was dated to the 4th-2nd centuries BC in China^46^, so the sticks in Xiaohe Cemetery could provide information for the development of early painting tool.

The cosmetic sticks were found in several archaeological sites in Xinjiang. In tomb M11 of Suebeixi Cemeteries^47^ (5th -3rd centuries BC), a leather bag was unearthed with string, comb, stone stick and red, black and white pigments and all of these were considered as a set of cosmetic implements. The stone stick had smooth surface and conical shape. A conical stick of 6.5 cm in length and 0.9 cm in diameter was found with a leather bag having brown powder in Sangeqiao Cemetery^48^ (4th -2nd centuries BC). The similar sticks and pigments were found in Zagunluk Cemetery^49^ (7th centuries BC -1st centuries AD), Baileqier Cemetery^50^ (cal. 2500–2700BP), Chawuhu Valley Cemetery^51^ (cal. 3000–2500BP) and so on. Since these sticks were usually unearthed with pigments, they were generally considered to be used for cosmetic, and sticks, pigment and/or leather bag were treated as a set of implements which was helpful to judge the gender of tomb occupier due to they were always buried with the female. These sticks share the similar shape and materials^23^: rod-shaped or conical and made of stone or wood (Fig. S3), which were distinct from those of Xiaohe Cemetery. Although cosmetic sticks and leather bags in Xiaohe Cemetery and other sites had the similar usage, the materials, shape and production of cosmetic sticks change greatly: more exquisite, suitable shape like today crayon and more wear-resistant materials.

More importantly, the cattle heart sticks had obvious religious significance. According to the excavated report, many of the relics in Xiaohe Cemetery were red-painted notably, such as the horn of the ox, the end of the arrow and the stand wooden pillar in front of the coffin^24^,^32^. The red colour was usually considered as a symbol of the worship for the blood^52^. The heart is one of the most important organs in cattle, which is the centre of the blood circulation. Using cattle heart as the painting tools might show that the worship of the blood was the mainly signature of the social religious culture. In addition, the stand wooden pillar in front of the coffin was considered as the sign of the reproduction worship. So using cattle heart as tools to paint red on human face and objects such as stand wooden pillar and wooden phallus indicated that painting red was a sacred and significant religious behaviour for Xiaohe people. Because the cosmetic sticks were in the leather bags buried with the female mummy, it is deduced that the woman carried out painting or making up with cosmetic sticks, which implied that woman played a special role in religious activities.

Plenty of evidences provide the wide use of cattle in Xiaohe culture, including cattle skulls tied to the wooden grave markers, cattle hide wrapping the coffins, cattle hide boots, buried cattle ears etc^24^,^32^. Xiaohe people widely used cattle’s products: sacrifice in the religious practices; a source of food, besides the meat, proteomic analysis indicated that milk and kefir cheese played an important role of Xiaohe people’ diet^30^,^53^; a kind of cloth; even the cow dung was used as a source of fertilizer or fuel^54^; and to manufacture animals glue^55^. Moreover, as indicated in this study, the heart of cattle was used as a cosmetic stick–a special painting tool.

Furthermore, Xiaohe Cemetery located in the desert where the output of hematite was never reported, so the source of the hematite was a significant issue to consider. Clarifying the details of communication areas and routes was required to do more research such as the distribution of hematite in Xinjiang, the migration of population among different culture and so on. All of these are the focus of the further work.

In this study, we identify that the red cosmetic sticks unearthed in Xiaohe Cemetery were made of cattle heart and the red pigment was hematite. Painting red and utility of cattle heart was the reflection of the society culture and worship in Xiaohe culture. More importantly, the women acted as the painter involved in religious activities. This work reveals the characterization of the cosmetic sticks used in ancient Xinjiang in China almost 3500 years ago, and the results provided a new perspective to understand the utilizations of cattle of Xiaohe people. Furthermore, it could broaden our understanding of the applications of the animal heart in prehistoric times.

## Methods

### SR-μCT scanning

The sticks were scanned by SR-μCT at the Shanghai Synchrotron Radiation Facility (SSRF), 13W, Shanghai, China. The parallel SR X-ray with the width of 2 cm and height of 4 mm was directed at the object with a source energy setting of 18 keV. The space resolution of the CCD detector was 9 μm. The data were analyzed by Mimics 12 software.

### FTIR analysis

FTIR analysis was employed to characterize major constituent of the painting and guide further analysis. The samples were analysed as KBr micro pellets with a Nicolet 6700 (Thermo Scientific) FTIR spectrometer working in a transmission mode. Spectra were acquired over the range of 4000–400 cm^−1^ using a resolution of 4 cm^−1^, with 32 scans per spectrum. The software OMNIC 8.0 was applied to deal with the data.

### Raman analysis

Raman analysis was performed on Laser Micro-Raman Spectrometer (JY XploRA, France). In the dark room and at room temperature, after being calibrated with a single crystal Si wafer sample, the sample excitation was performed with a 785 nm laser and focused through a 50× objective lens. The diffraction grating was 1200 g/mm and the width of the entrance slit of spectrometer was 100 μm. Spectra of the samples were recorded with a resolution of 2 cm^−1^ and three accumulations of 20 s each in the range 100–1000 cm^−1^ using the NGS LabSpec software.

### Proteomic analysis

For each archaeological sample a subsample of 20 mg was suspended in 100 μL of extracting solution (Tris/HCl, pH 8.0, 10% SDS, 10 mM DTT and 0.0025% bromphenol blue). After being subjected to ultrasonic baths (3 × 15 min), the sample was incubated for 1 h at 56 °C. Then the sample was subjected to ultrasonic baths again for 15 min and centrifuged for 15 min at 12,000 g.

45 μL of the supernatant of each sample was mixed with 5 μL of glycerol, heated at 95 °C for 5 min. The extracting solution was loaded onto the gel (SDS-PAGE, sodium dodecyl sulfate polyacrylamide gel electrophoresis) with 25 μL each well. The electrophoresis apparatus was connected to a 200 V power. The gel with sample was stained by the solution (0.25% Coomassie Blue w/v, 50% ethanol, 10% acetic acid) and destained by the solution (25% ethanol, 8% acetic acid) until protein bands visible.

The gel with protein bands was cut into small pieces and washed by distilled water three times. Then the gel pieces were destained with 50% acetonitrile/25 mM NH_4_HCO_3_ and alkylated in the dark with 50 mM iodoacetamide for 30 min. After being washed with 25 mM NH_4_HCO_3_ buffer twice, the gel pieces of each sample were immersed in 12.5 ng/μL trypsin solutions, approximate 50–80 μL.And put in the microwave oven at 850W for 1 min for the digestion.

0.1% formic acid was added to dissolve the digested samples. Then the samples were analysed by RP C18 capillary LC column from Michrom Bioresources (100 μm × 150 mm, 3 μm). The parameters of LTQ Orbitrap Velos mass spectrometer was: 10 data-dependent HCD MS/MS scans per every full scan; a resolution of 30,000 and 30% normalized collision energy; charge state screening (excluding precursors with unknown charge state or +1 charge state); internal mass calibration (445.120025 ion as lock mass with a target lock mass abundance of 0%) and dynamic exclusion (exclusion size list 500, exclusion duration 30 s).

The MS/MS spectra were searched against the NCBInr (20093899 sequences; 6882348701 residues) by Mascot software version 2.4.1 (Matrix Science, UK). Trypsin was selected as the proteolytic enzyme and two missed cleavages were allowed. Carbamidomethylation (C) was selected as fixed modification. Deamidation (N and Q) and Dioxidation (M) were selected as variable amino-acid modifications. A peptide tolerance of 5 ppm and a product ion tolerance of 0.05 Da were used in the searches and the peptides were filtered with significance threshold p < 0.05 and ions score cut-off 13.

## Additional Information

**How to cite this article**: Mai, H. *et al.* Characterization of cosmetic sticks at Xiaohe Cemetery in early Bronze Age Xinjiang, China. *Sci. Rep.* **6**, 18939; doi: 10.1038/srep18939 (2016).

## Supplementary Material

Supplementary Information

## Figures and Tables

**Figure 1:**
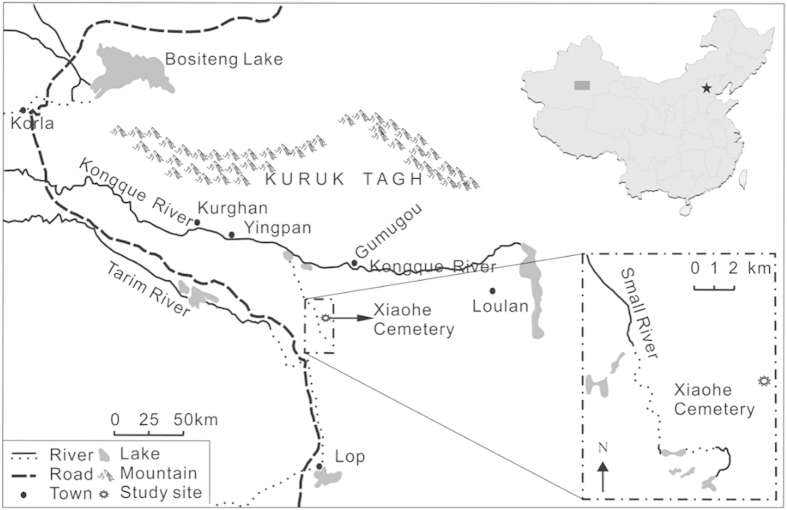
The location of Xiaohe Cemetery (modified from Li, 2013^56^).

**Figure 2:**
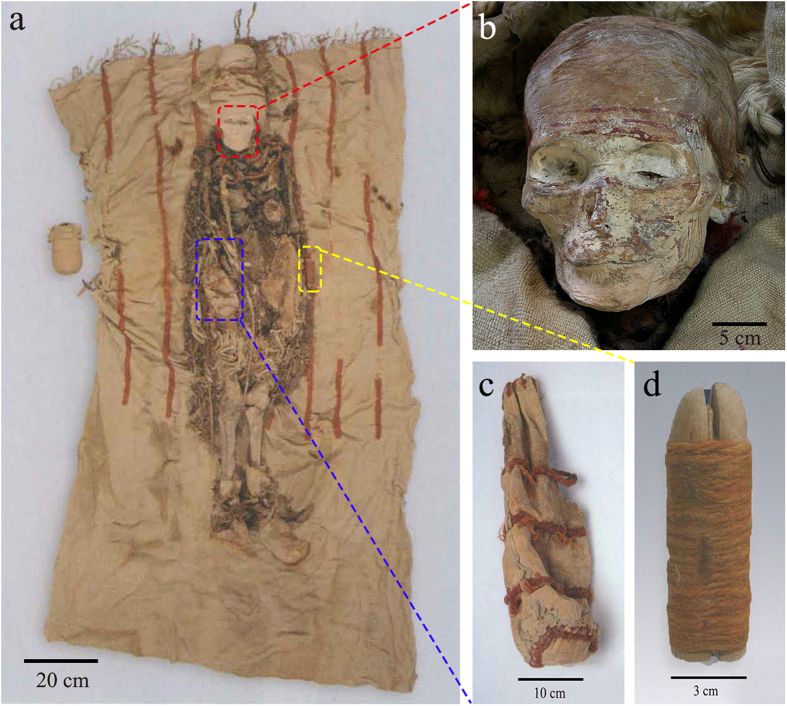
The stuffs discovered in tomb M13: (**a**) the front view of the female mummy with her funerary objects. The leather bag was on the right side of her waist (marked by a blue box), the wooden genitalia were on the left side (marked by a yellow box) and the wooden comb was under the right of the buttocks; (**b**) the red lines painted on the forehead of the mummy; (**c**) the leather bag; (**d**) the wooden genitalia.

**Table 1:** Identified protein and specific peptides in sticks.

Sample	Identified proteins	Accession number	Seq. Cov. [%]	Matching Peptides	Unique Peptides^*^	Position	Score	Species
Stick 1 (from M17)	myosin-7	gi|41386711	19	38	–	–	–	–
	Predicted: myosin-6	gi|297479068	13	26	–	–	–	–
	myosin light chain 3	gi|270483786	36	6	AAAAPAPAPAPPPAPEPSK	19–37	49	*Bos taurus, Bison bison bison, Bubalus bubalis*
	alpha-actinin-2	gi|77736221	34	28	–	–	–	–
	Predicted: Low Quality Protein: titin	gi|297465038	1	43	–	–	–	–
	desmin	gi|2959452	37	16	–	–	–	–
	myosin-binding protein C, cardiac-type	gi|115495853	15	19	SIFTVEGAER	728–737	45	*Bos taurus, Bison bison bison, Bos mutus*
					AHNLAGAGPPVTTK	941–954	89	*Bos taurus, Bos mutus*
	Serum albumin	gi|1351907	33	21	LVNELTEFAK	66–75	74	*Bos taurus, Bison bison bison, Bubalus bubalis, Bos mutus*
					TCVADESHAGCEK	76–88	74	*Bos taurus, Bison bison bison, Bos mutus*
					DAFLGSFLYEYSR	347–359	42	*Bos indicus, Bos taurus, Bison bison bison, Bubalus bubalis, Bos mutus*
					RPCFSALTPDETYVPK	508–523	51	*Bos indicus, Bos taurus, Bison bison bison, Bubalus bubalis, Bos mutus*
	myoglobin	gi|27806939	50	6	HPSDFGADAQAAMSK	120–134	47	*Bos taurus, Bubalus bubalis*
	hemoglobin alpha chain	gi|6006425	30	4	VGGHAAEYGAEALER	18–32	82	*Bos taurus, Bison bison bison, Bubalus bubalis, Bos mutus, Bison bonasus, Bos grunniens*
	collagen alpha-1(VI) chain precursor	gi|219804724	9	10	AAEYDVVFGER	990–1000	39	*Bos taurus, Bison bison bison, Bubalus bubalis, Bos mutus*
	Predicted: collagen alpha-3(VI) chain isoform 4	gi|297473452	9	24	–	–	–	–
Stick 2 (from M22)	myosin-7	gi|41386711	15	35	–	–	–	–
	tropomyosin alpha-1 chain	gi|61888866	11	4	–	–	–	–
	tropomyosin 4-like	gi|296486595	15	4	IQLVEEELNRAQER	56–69	39	*Bos taurus, Bison bison bison, Bubalus bubalis, Bos mutus*
	Predicted: Low Quality Protein: titin	gi|297465038	<1	31	NGVVLESSDK	2730–2739	19	*Bos taurus*
	desmin	gi|2959452	11	5	–	–	–	–
	myosin-binding protein C, cardiac-type	gi|115495853	4	5	–	–	–	–
	Serum albumin	gi|1351907	31	21	YICDNQDTISSK	286–297	25	*Bos indicus, Bos taurus, Bison bison bison, Bubalus bubalis, Bos mutus*
					DAIPENLPPLTADFAEDK	319–336	64	*Bos indicus, Bos taurus*
	Hemoglobin subunit alpha-2	gi|122286	40	5	VGGHAAEYGAEALER	17–31	61	*Bos taurus, Bos grunniens, Bison bison bison, Bos mutus, Bison bonasus*
	collagen, type VI, alpha 3-like isoform 3	gi|296488813	<1	8	–	–	–	–
