# Supplementary material for: Characterization of cosmetic sticks at Xiaohe Cemetery in early Bronze Age Xinjiang, China
Source: Sci Rep. 2016 Jan 28;6:18939. doi: 10.1038/srep18939 (PMC4730899; doi:10.1038/srep18939)
Supplement: Supplementary Information [file srep18939-s1.pdf]

Supplementary Information

Characterization of cosmetic sticks at Xiaohe Cemetery in early Bronze Age Xinjiang, China

Huijuan Mai[1, 2], Yimin Yang[2,*], Idelisi Abuduresule[3], Wenying Li[3], Xingjun Hu[3], Changsui Wang[2]

1. Key Laboratory of Vertebrate Evolution and Human Origins of Chinese Academy of Sciences, Institute of Vertebrate Paleontology and Paleoanthropology, Chinese Academy of Sciences, Beijing 100044, People's Republic of China

2. Department of Archaeology and Anthropology, University of Chinese Academy of Sciences, Beijing 100049, People's Republic of China

3. Xinjiang Cultural Relics and Archaeology Institute, Ürümchi 830000, People's Republic of China

Figure S1 FTIR spectra of the inside material of stick1 (a) and stick2 (b)

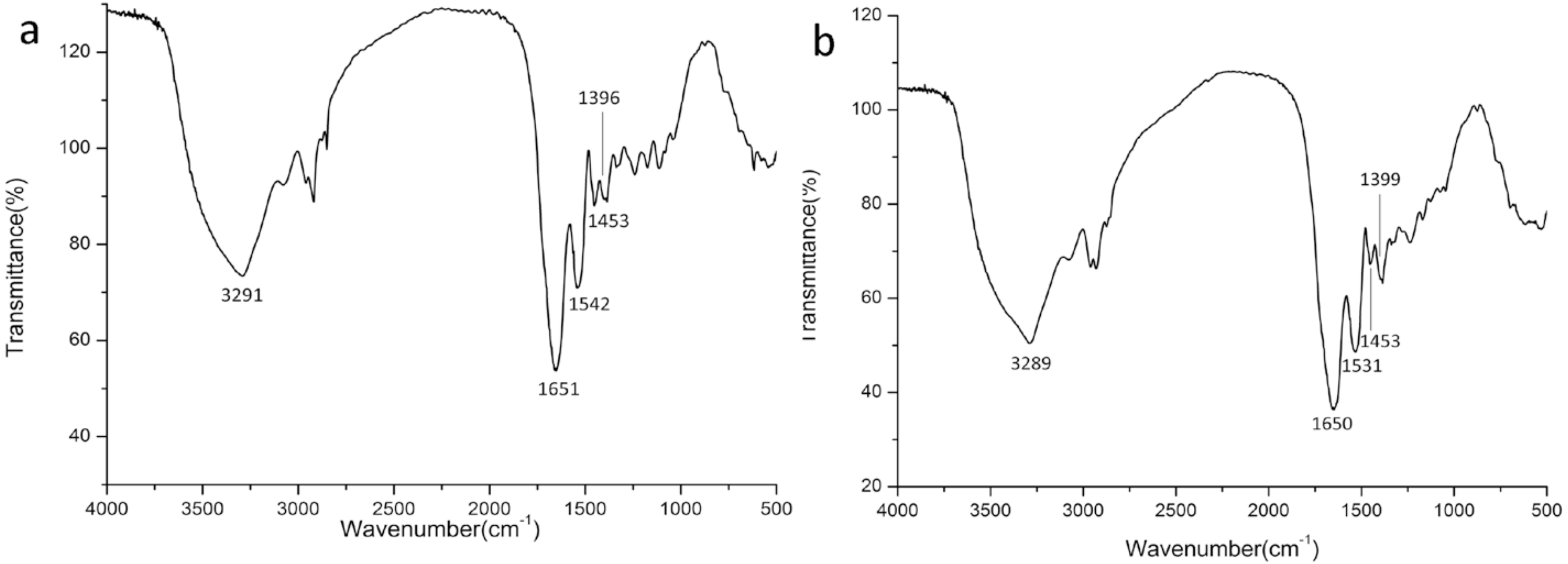

Figure S2 The gel figure of stick1 (a) and stick2 (b)

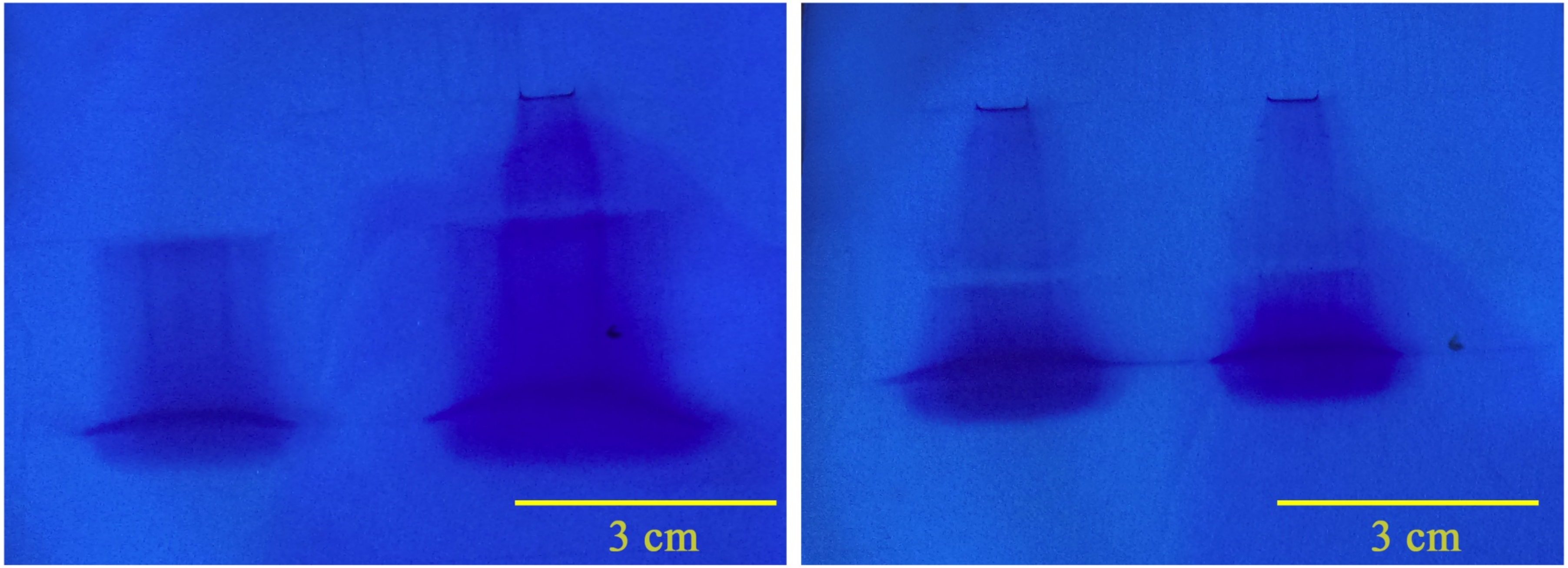

Figure S3 Sketches of sticks unearthed in archaeological sites in Xinjiang

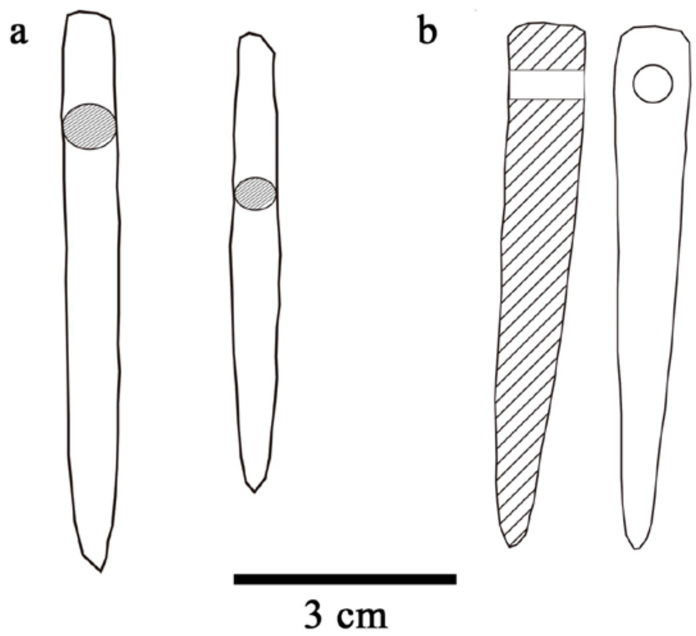

Figure S3: a) stick found in Baileqier Cemetery M206; b) stick found in Chawuhu Valley Cemetery M221

Table S1 Details of peptides of stick1 (from M17) identified by searching against the NCBInr database

<table>
<tr><td>1</td><td colspan="10">gi|41386711 Mass: 223889 Score: 585 Sequences: 38 emPAI: 0.16
myosin-7 [Bos taurus]</td></tr>
<tr><th></th><th>Query</th><th>Observed</th><th>Mr(expt)</th><th>Mr(calc)</th><th>Delta</th><th>Miss</th><th>Score</th><th>Expect</th><th>Rank</th><th>Peptide</th></tr>
<tr><td></td><td>140</td><td>374.2428</td><td>746.4711</td><td>746.4691</td><td>0.0021</td><td>0</td><td>(20)</td><td>17</td><td>4</td><td>R.VIFQLK.A 139</td></tr>
<tr><td></td><td>145</td><td>374.7346</td><td>747.4546</td><td>747.4531</td><td>0.0015</td><td>0</td><td>23</td><td>11</td><td>6</td><td>R.VIFQLK.A</td></tr>
<tr><td></td><td>286</td><td>395.2398</td><td>788.4650</td><td>788.4643</td><td>0.0006</td><td>0</td><td>26</td><td>10</td><td>9</td><td>K.SLQSLLK.D</td></tr>
<tr><td></td><td>485</td><td>415.7301</td><td>829.4456</td><td>829.4446</td><td>0.0009</td><td>0</td><td>16</td><td>80</td><td>2</td><td>R.IHFGATGK.L</td></tr>
<tr><td></td><td>870</td><td>442.2390</td><td>882.4634</td><td>882.4599</td><td>0.0034</td><td>0</td><td>56</td><td>0.0054</td><td>1</td><td>R.ILYGDFR.Q 863 864 865 866 867 868 869</td></tr>
<tr><td></td><td>908</td><td>445.2519</td><td>888.4892</td><td>888.4916</td><td>-0.002</td><td>0</td><td>(34)</td><td>1.6</td><td>1</td><td>R.INATLETK.Q</td></tr>
<tr><td></td><td>919</td><td>445.7461</td><td>889.4776</td><td>889.4756</td><td>0.0020</td><td>0</td><td>40</td><td>0.41</td><td>1</td><td>R.INATLETK.Q</td></tr>
<tr><td></td><td>1145</td><td>462.7565</td><td>923.4985</td><td>923.4964</td><td>0.0021</td><td>0</td><td>34</td><td>1</td><td>1</td><td>R.SLSTELFK.L</td></tr>
<tr><td></td><td>1516</td><td>488.2462</td><td>974.4779</td><td>974.4743</td><td>0.0037</td><td>0</td><td>28</td><td>5.2</td><td>2</td><td>R.CIIPNETK.S</td></tr>
<tr><td></td><td>1562</td><td>490.7578</td><td>979.5011</td><td>979.4988</td><td>0.0023</td><td>0</td><td>24</td><td>12</td><td>1</td><td>R.STHPHFVR.C</td></tr>
<tr><td></td><td>1867</td><td>509.2720</td><td>1016.5294</td><td>1016.5073</td><td>0.0221</td><td>0</td><td>(31)</td><td>7</td><td>1</td><td>R.CNGVLEGIR.I</td></tr>
<tr><td></td><td>1873</td><td>509.7587</td><td>1017.5028</td><td>1017.4913</td><td>0.0115</td><td>0</td><td>40</td><td>0.38</td><td>1</td><td>R.CNGVLEGIR.I 1874</td></tr>
<tr><td></td><td>1982</td><td>516.2933</td><td>1030.5720</td><td>1030.5658</td><td>0.0061</td><td>0</td><td>48</td><td>0.055</td><td>1</td><td>K.EALISQLTR.G</td></tr>
<tr><td></td><td>2550</td><td>547.2837</td><td>1092.5528</td><td>1091.5499</td><td>1.0030</td><td>0</td><td>23</td><td>21</td><td>1</td><td>R.AQLEFNQIK.A</td></tr>
<tr><td></td><td>2758</td><td>558.7556</td><td>1115.4966</td><td>1115.4892</td><td>0.0074</td><td>0</td><td>33</td><td>0.73</td><td>1</td><td>R.MFNWMVTR.I</td></tr>
<tr><td></td><td>2764</td><td>559.2449</td><td>1116.4751</td><td>1116.4732</td><td>0.0019</td><td>0</td><td>(32)</td><td>0.58</td><td>1</td><td>R.MFNWMVTR.I 2765</td></tr>
<tr><td></td><td>3115</td><td>577.2989</td><td>1152.5832</td><td>1152.5887</td><td>-0.005</td><td>0</td><td>(16)</td><td>70</td><td>1</td><td>K.NALAHALQSAR.H</td></tr>
<tr><td></td><td>204</td><td>385.2072</td><td>1152.5997</td><td>1151.6047</td><td>0.9950</td><td>0</td><td>39</td><td>0.86</td><td>1</td><td>K.NALAHALQSAR.H</td></tr>
<tr><td></td><td>3886</td><td>609.8310</td><td>1217.6474</td><td>1216.6485</td><td>0.9989</td><td>0</td><td>89</td><td>4.9e-006</td><td>1</td><td>K.AGLLGLLEEMR.D 3873 3874 3875</td></tr>
</table>

	4116	618.8124	1235.6102	1234.6194	0.9909	0	16	99	1	K.GQNVQQVVYAK.G
	4129	619.3043	1236.5940	1235.6034	0.9906	0	(15)	96	1	K.GQNVQQVVYAK.G
	4228	623.3327	1244.6508	1244.6500	0.0008	0	59	0.004	1	R.DIDDLELTLAK.V
	4784	645.8258	1289.6370	1289.6364	0.0006	0	61	0.003	1	K.GSSFQTVSALHR.E 4783
	4785	430.8893	1289.6462	1289.6364	0.0098	0	(17)	70	1	K.GSSFQTVSALHR.E
	5039	657.8819	1313.7493	1313.7442	0.0051	1	47	0.035	1	R.LQDLVDKLQLK.V
	5085	659.8559	1317.6972	1317.6928	0.0043	1	20	46	1	R.LEAQTRPFDLK.K 5084
	5290	669.8387	1337.6628	1337.6649	-0.002	1	38	0.53	1	K.EIALMKEEFGR.L
	5361	449.2419	1344.7038	1344.6885	0.0154	1	64	0.0012	1	R.ADIAESQVNKLR.A 5359
	5429	676.8232	1351.6318	1351.6264	0.0054	0	20	25	1	K.LTGAIMHFGNMK.F
	5631	686.4062	1370.7978	1370.7558	0.0420	0	(21)	12	2	R.DSLLIIQWNIR.A
	5647	686.8802	1371.7458	1371.7398	0.0061	0	39	0.38	1	R.DSLLIIQWNIR.A 5646 5648 5649 5650 5653 5654 5664
	6115	709.3617	1416.7089	1416.7096	-0.000	0	(32)	5.1	1	K.LAEQELIETSER.V
	6136	709.8558	1417.6971	1417.6936	0.0035	0	96	8.6e-007	1	K.LAEQELIETSER.V
	6390	722.3786	1442.7427	1442.7252	0.0175	0	(70)	0.0008	1	R.NNLLQAELEELR.A
	6403	722.8683	1443.7221	1442.7252	0.9969	0	(44)	0.13	1	R.NNLLQAELEELR.A
	6404	722.8729	1443.7312	1443.7092	0.0219	0	73	0.00017	1	R.NNLLQAELEELR.A
	6596	733.3971	1464.7796	1464.7711	0.0085	0	84	2.5e-005	1	K.LASADIETYLLEK.S 6588 6589 6590 6591 6592 6593 6594 6595 6598 6599 6600 6607 6608 6609 6611 6614 6617
	6776	742.3574	1482.7002	1482.6950	0.0052	1	42	0.17	1	R.KVQHELDEAEER.A
	6822	744.8632	1487.7118	1487.6991	0.0127	0	61	0.0021	1	R.IEELEEELEAER.T
	6973	753.3739	1504.7332	1504.7256	0.0075	0	33	4.5	1	R.ENQSILITGESGAGK.T
	7074	506.2615	1515.7627	1515.7569	0.0058	0	(29)	3.9	1	K.LLGSLDIDHNQYK.F

	7084	506.5892	1516.7457	1516.7409	0.0048	0	74	0.00013	1	K.LLGSLDIDHNQYK.F
	7155	510.2884	1527.8433	1527.8409	0.0024	1	26	5	1	R.RDSLLIIQWNIR.A
	7196	767.8834	1533.7522	1533.7522	-0.000	0	15	1.2e+002	1	R.VVDSLQTSLDAETR.S
	7210	768.4231	1534.8316	1534.8508	-0.019	0	(53)	0.012	1	R.VIQYFAVIAAIGDR.S 7207 7212 7213 7217 7223
	7219	768.9270	1535.8395	1535.8348	0.0047	0	112	1.2e-008	1	R.VIQYFAVIAAIGDR.S 7218 7220 7221 7222 7224 7225 7226 7227 7228 7229 7236
	7266	771.3910	1540.7675	1540.7562	0.0114	0	66	0.00074	1	R.DYHIFYQILSNK.K
	7267	771.3951	1540.7756	1540.7562	0.0195	0	(15)	84	1	R.DYHIFYQILSNK.K
	7275	771.8839	1541.7532	1541.7402	0.0130	0	(61)	0.0021	1	R.DYHIFYQILSNK.K
	7321	516.2734	1545.7985	1545.7886	0.0099	1	19	40	1	R.KLAEQELIETSER.V 7320
	7783	539.9486	1616.8239	1616.8192	0.0047	1	39	0.44	1	K.AGLLGLLEEMRDER.L 2417
	7784	809.4201	1616.8257	1616.8192	0.0065	1	(16)	94	1	K.AGLLGLLEEMRDER.L
	8215	564.9621	1691.8646	1691.8665	-0.001	0	67	0.00062	1	K.SPGVIDNPLVMHQLR.C
	8408	871.4594	1740.9043	1740.9046	-0.000	0	79	3.3e-005	1	R.ILNPAAIPEGQFIDSR.K 8409 8410 8414
	8413	581.3105	1740.9096	1740.9046	0.0049	0	(50)	0.029	1	R.ILNPAAIPEGQFIDSR.K 8415
	8420	871.9547	1741.8949	1741.8886	0.0062	0	(46)	0.078	1	R.ILNPAAIPEGQFIDSR.K
	8421	581.6392	1741.8957	1741.8886	0.0071	0	(45)	0.095	1	R.ILNPAAIPEGQFIDSR.K
2	gi|297479068 Mass: 223466 Score: 349 Sequences: 26 emPAI: 0.12 PREDICTED: myosin-6 [Bos taurus]									
	Query	Observed	Mr(expt)	Mr(calc)	Delta	Miss	Score	Expect	Rank	Peptide
	140	374.2428	746.4711	746.4691	0.0021	0	(20)	17	4	R.VIFQLK.A 139
	145	374.7346	747.4546	747.4531	0.0015	0	23	11	6	R.VIFQLK.A
	485	415.7301	829.4456	829.4446	0.0009	0	16	80	2	R.IHFGATGK.L
	870	442.2390	882.4634	882.4599	0.0034	0	56	0.0054	1	R.ILYGDFR.Q 863 864 865 866 867 868 869
	1145	462.7565	923.4985	923.4964	0.0021	0	34	1	1	R.SLSTELFK.L

	1867	509.2720	1016.5294	1016.5073	0.0221	0	(31)	7	1	R.CNGVLEGIR.I
	1873	509.7587	1017.5028	1017.4913	0.0115	0	40	0.38	1	R.CNGVLEGIR.I 1874
	1982	516.2933	1030.5720	1030.5658	0.0061	0	48	0.055	1	K.EALISQLTR.G
	2550	547.2837	1092.5528	1091.5499	1.0030	0	23	21	1	R.AQLEFNQIK.A
	2758	558.7556	1115.4966	1115.4892	0.0074	0	33	0.73	1	K.MFNWMVTR.I
	2764	559.2449	1116.4751	1116.4732	0.0019	0	(32)	0.58	1	K.MFNWMVTR.I 2765
	3115	577.2989	1152.5832	1152.5887	-0.005	0	(16)	70	1	K.NALAHALQSAR.H
	204	385.2072	1152.5997	1151.6047	0.9950	0	39	0.86	1	K.NALAHALQSAR.H
	3886	609.8310	1217.6474	1216.6485	0.9989	0	89	4.9e-006	1	K.AGLLGLLEEMR.D 3873 3874 3875
	4228	623.3327	1244.6508	1244.6500	0.0008	0	59	0.004	1	K.DIDDLELTLAK.V
	4784	645.8258	1289.6370	1289.6364	0.0006	0	61	0.003	1	K.GSSFQTVSALHR.E 4783
	4785	430.8893	1289.6462	1289.6364	0.0098	0	(17)	70	1	K.GSSFQTVSALHR.E
	5039	657.8819	1313.7493	1313.7442	0.0051	1	47	0.035	1	R.LQDLVDKLQLK.V
	5361	449.2419	1344.7038	1344.6885	0.0154	1	64	0.0012	1	R.ADIAESQVNKLR.A 5359
	5429	676.8232	1351.6318	1351.6264	0.0054	0	15	84	2	K.LTGAIMHYGNMK.F
	6115	709.3617	1416.7089	1416.7096	-0.000	0	(32)	5.1	1	K.LAEQELIETSER.V
	6136	709.8558	1417.6971	1417.6936	0.0035	0	96	8.6e-007	1	K.LAEQELIETSER.V
	6390	722.3786	1442.7427	1442.7252	0.0175	0	(70)	0.0008	1	R.NNLLQAELEELR.A
	6403	722.8683	1443.7221	1442.7252	0.9969	0	(44)	0.13	1	R.NNLLQAELEELR.A
	6404	722.8729	1443.7312	1443.7092	0.0219	0	73	0.00017	1	R.NNLLQAELEELR.A
	6596	733.3971	1464.7796	1464.7711	0.0085	0	84	2.5e-005	1	K.LASADIETYLLEK.S 6588 6589 6590 6591 6592 6593 6594 6595 6598 6599 6600 6607 6608 6609 6611 6614 6617
	6776	742.3574	1482.7002	1482.6950	0.0052	1	42	0.17	1	R.KVQHELDEAEER.A
	6822	744.8632	1487.7118	1487.6991	0.0127	0	61	0.0021	1	R.IEELEEELEAER.T

	7074	506.2615	1515.7627	1515.7569	0.0058	0	(29)	3.9	1	K.LLGSLDIDHNQYK.F
	7084	506.5892	1516.7457	1516.7409	0.0048	0	74	0.00013	1	K.LLGSLDIDHNQYK.F
	7196	767.8834	1533.7522	1533.7522	-0.000	0	15	1.2e+002	1	R.VVDSLQTSLDAETR.S
	7266	771.3910	1540.7675	1540.7561	0.0114	0	66	0.00074	1	R.NYHIFYQILSNK.K
	7267	771.3951	1540.7756	1540.7561	0.0195	0	(15)	84	1	R.NYHIFYQILSNK.K
	7275	771.8839	1541.7532	1541.7402	0.0131	0	(59)	0.0038	2	R.NYHIFYQILSNK.K
	7321	516.2734	1545.7985	1545.7886	0.0099	1	19	40	1	R.KLAEQELIETSER.V 7320
	7783	539.9486	1616.8239	1616.8192	0.0047	1	39	0.44	1	K.AGLLGLLEEMRDER.L 2417
	7784	809.4201	1616.8257	1616.8192	0.0065	1	(16)	94	1	K.AGLLGLLEEMRDER.L
	8408	871.4594	1740.9043	1740.9046	-0.0003	0	79	3.3e-005	1	R.ILNPAAIPEGQFIDSR.K 8409 8410 8414
	8413	581.3105	1740.9096	1740.9046	0.0049	0	(50)	0.029	1	R.ILNPAAIPEGQFIDSR.K 8415
	8420	871.9547	1741.8949	1741.8886	0.0062	0	(46)	0.078	1	R.ILNPAAIPEGQFIDSR.K
	8421	581.6392	1741.8957	1741.8886	0.0071	0	(45)	0.095	1	R.ILNPAAIPEGQFIDSR.K
**3**	**gi|270483786 Mass: 22096 Score: 125 Sequences: 6 emPAI: 0.74 myosin light chain 3 [Bos taurus]**									
	1464	**484.2840**	**966.5534**	**966.5498**	**0.0035**	**0**	**60**	**0.0014**	**1**	**R.HVLATLGEK.L**
	1714	**499.7557**	**997.4969**	**997.4869**	**0.0100**	**0**	**55**	**0.0077**	**1**	**K.EAFTLFDR.T** 1713
	4678	**641.3154**	**1280.6162**	**1280.6183**	**-0.0021**	**0**	**(39)**	**0.39**	**1**	**K.ITYGQCGDVLR.A**
	4685	**641.8098**	**1281.6050**	**1281.6023**	**0.0027**	**0**	**73**	**0.00013**	**1**	**K.ITYGQCGDVLR.A** 4686 4687
	5906	**699.3778**	**1396.7410**	**1396.7310**	**0.0100**	**0**	**(57)**	**0.011**	**1**	**R.ALGQNPTQAEVLR.V**
	5912	**699.8662**	**1397.7178**	**1397.7150**	**0.0028**	**0**	**(69)**	**0.00035**	**1**	**R.ALGQNPTQAEVLR.V** 5913
	5921	**700.3583**	**1398.7021**	**1398.6990**	**0.0031**	**0**	**92**	**2e-006**	**1**	**R.ALGQNPTQAEVLR.V** 5919 5920 5934
	7009	**755.3805**	**1508.7465**	**1508.7399**	**0.0067**	**0**	**(13)**	**1.5e+002**	**1**	**K.IEFTPEQIEEFK.E**
	7013	**755.8762**	**1509.7378**	**1509.7239**	**0.0139**	**0**	**42**	**0.21**	**1**	**K.IEFTPEQIEEFK.E**
	8261	**854.4545**	**1706.8944**	**1706.8991**	**-0.0047**	**0**	**49**	**0.038**	**1**	**K.AAAAPAPAPAPPPAPEPSK.E**

4	gi|77736221 Mass: 104284 Score: 371 Sequences: 28 emPAI: 0.37<br>alpha-actinin-2 [Bos taurus]									
	**Query**	**Observed**	**Mr(expt)**	**Mr(calc)**	**Delta**	**Miss**	**Score**	**Expect**	**Rank**	**Peptide**
	57	361.7151	721.4157	721.4123	0.0034	0	16	91	1	K.HLDIPK.M
	75	363.7296	725.4447	724.4595	0.9852	0	22	5.8	1	K.QLVPIR.D
	367	404.2248	806.4351	806.4286	0.0065	1	23	15	1	K.KLEDFR.D
	854	440.7428	879.4710	879.4702	0.0009	0	55	0.0092	1	K.ALDYIASK.G
	1456	483.7665	965.5184	965.5182	0.0003	0	66	0.00073	1	K.VIQSYSIR.I 1457 1461
	1458	483.7689	965.5232	964.5342	0.9890	0	(44)	0.093	1	K.VIQSYSIR.I
	1672	497.2393	992.4641	992.4464	0.0177	0	29	3.9	1	R.ASFNHFDR.R
	1965	515.2747	1028.5349	1028.5291	0.0058	0	22	16	2	R.TIPWLENR.T
	3330	586.8026	1171.5907	1171.5833	0.0074	0	45	0.086	1	K.VLAVNQENER.L
	3353	587.7890	1173.5635	1173.5513	0.0122	0	(28)	4.3	1	K.VLAVNQENER.L
	3362	588.3003	1174.5860	1174.5805	0.0056	0	52	0.022	1	K.EGLLLWCQR.K
	3365	588.3074	1174.6002	1173.5965	1.0038	0	(17)	71	1	K.EGLLLWCQR.K
	4037	615.3500	1228.6854	1228.6815	0.0039	0	75	8e-005	1	R.LASELLEWIR.R 4036 4038 4053
	4198	622.3251	1242.6357	1242.6357	0.0001	1	26	7.1	1	R.HRPDLIDYSK.L
	4270	625.8044	1249.5942	1249.5761	0.0181	0	66	0.00068	1	K.MVSDIAGAWQR.L 4269
	4886	650.3101	1298.6057	1298.5990	0.0067	0	71	0.00017	1	K.DYESSTLTEVR.A
	5080	440.2022	1317.5848	1317.5772	0.0076	0	60	0.0016	1	K.HTNYTMEHIR.V
	5104	660.8097	1319.6048	1317.5772	2.0276	0	(22)	13	1	K.HTNYTMEHIR.V
	5635	686.4072	1370.7998	1370.7922	0.0076	0	53	0.0091	1	R.VGWELLLTTIAR.T 5626 5627 5628 5630 5631 5632 5634 5650 5656 5657
	5663	687.3685	1372.7224	1372.7085	0.0139	0	(20)	75	1	R.QSILAIQNEVEK.V
	5671	687.8583	1373.7020	1373.6925	0.0095	0	32	2.3	1	R.QSILAIQNEVEK.V

	5823	694.8949	1387.7751	1387.7745	0.0007	0	61	0.0018	1	K.LMLLLEVISGER.L 5816 5818 5819 5820 5821 5822 5824 5825 5826 5827 5828 5829 5839 5840 5841
	6126	709.3823	1416.7501	1416.7460	0.0041	0	(77)	0.00014	1	R.TINEVETQILTR.D 6116
	6138	709.8748	1417.7351	1417.7300	0.0051	0	94	1.4e-006	1	R.TINEVETQILTR.D 6139 6140
	6461	725.8555	1449.6965	1449.6922	0.0043	1	23	27	1	R.ISNRPAFMPSEGK.M
	6555	732.3416	1462.6687	1462.6650	0.0038	0	21	16	1	R.ELPPDQAQYCIK.R 6557
	7243	769.8868	1537.7591	1537.7511	0.0080	0	99	3.8e-007	1	R.FAIQDISVEETSAK.E 7242
	7244	769.8902	1537.7658	1536.7671	0.9987	0	(57)	0.0068	1	R.FAIQDISVEETSAK.E
	7293	772.4168	1542.8190	1542.8141	0.0049	0	36	0.72	1	K.LVSIGAEEIVDGNVK.M 7306
	8099	557.6413	1669.9020	1669.8927	0.0093	0	47	0.048	1	K.LLETIDQLHLEFAK.R
	8362	865.9911	1729.9676	1729.9614	0.0062	1	(61)	0.0011	1	R.ILASDKPYILAEELR.R
	8363	577.6634	1729.9683	1729.9614	0.0069	1	110	1.4e-008	1	R.ILASDKPYILAEELR.R
	8461	877.9186	1753.8226	1753.8006	0.0220	0	113	2.6e-008	1	R.ETADTDTAEQVIASFR.I
	8495	881.9366	1761.8587	1760.8257	1.0330	0	26	8.3	1	R.QFAAQANAIGPWIQNK.M
	8516	884.4447	1766.8748	1766.8687	0.0062	0	125	1.1e-009	1	R.ISSSNPYSTVTVDEIR.S
	8939	633.3368	1896.9885	1895.9952	0.9933	1	26	5.9	1	K.QLVPIRDQSLQEELAR.Q
5	gi|297465038 Mass: 3741571 Score: 74 Sequences: 43 emPAI: 0.00<br>PREDICTED: LOW QUALITY PROTEIN: titin [Bos taurus]									
	Query	Observed	Mr(expt)	Mr(calc)	Delta	Miss	Score	Expect	Rank	Peptide
	6	350.7291	699.4436	699.4279	0.0157	0	24	6	1	R.LIGELR.L
	23	357.7307	713.4469	713.4436	0.0033	0	21	14	1	K.VVEVIR.D
	29	358.7284	715.4422	715.4381	0.0041	0	29	3.7	3	R.FPAIIR.G
	37	359.7086	717.4026	717.4021	0.0005	0	43	0.26	1	K.TLTDLR.Y
	40	359.7261	717.4376	717.4425	-0.0049	0	20	37	1	R.LYVPVK.G
	41	359.7356	717.4566	717.4537	0.0029	0	34	0.79	1	R.LFAIVR.G 42

	78	**364.2400**	**726.4655**	**726.4640**	**0.0015**	**0**	**24**	**3.4**	**1**	**K.VIVATPK.V**
	84	**365.2248**	**728.4351**	**728.4796**	**-0.0445**	**0**	**17**	**44**	**4**	**K.LVVTGLK.E**
	87	**365.2407**	**728.4669**	**728.4796**	**-0.0127**	**0**	**38**	**0.32**	**1**	**R.LIIAATK.L**
	128	**372.7433**	**743.4721**	**743.4694**	**0.0027**	**0**	**26**	**5.4**	**1**	**R.LFVPIR.G**
	151	**375.7219**	**749.4293**	**748.3868**	**1.0425**	**0**	**24**	**10**	**1**	**R.IFAENR.Y**
	266	392.7054	783.3962	783.3915	0.0047	0	30	1.6	1	R.FDIFSR.Y
	333	**400.7613**	**799.5080**	**798.4963**	**1.0117**	**0**	**16**	**35**	**8**	**R.LLIPSTR.M**
	358	**403.2479**	**804.4812**	**804.4858**	**-0.0046**	**0**	**28**	**3.6**	**1**	**R.LFVTGIR.L**
	395	**407.7174**	**813.4203**	**813.4174**	**0.0030**	**0**	**36**	**0.51**	**1**	**K.TPFFFR.V**
	851	**440.2476**	**878.4807**	**878.4862**	**-0.0055**	**0**	**22**	**19**	**1**	**K.DLVYTLR.I**
	971	**450.2359**	**898.4573**	**897.4344**	**1.0229**	**0**	**14**	**78**	**2**	**K.GNEYIFR.V**
	1067	**456.7703**	**911.5261**	**911.5189**	**0.0072**	**0**	**(15)**	**43**	**3**	**R.NAVGVSLPR.E**
	1073	**457.2451**	**912.4757**	**912.5029**	**-0.0272**	**0**	**31**	**1.8**	**1**	**R.NAVGVSLPR.E**
	1094	**458.7675**	**915.5204**	**915.5178**	**0.0026**	**0**	**34**	**1.5**	**1**	**R.IVPPSFTR.R**
	1144	**462.7435**	**923.4724**	**923.4535**	**0.0190**	**0**	**32**	**2**	**1**	**R.FECVLTR.E**
	1169	**464.7718**	**927.5291**	**927.5542**	**-0.0251**	**0**	**21**	**16**	**1**	**K.LSIPVPFR.A**
	1559	**490.2715**	**978.5284**	**978.5386**	**-0.0102**	**0**	**36**	**0.84**	**1**	**R.ITNYIVEK.R**
	1807	**505.8159**	**1009.6173**	**1009.6172**	**0.0001**	**0**	**21**	**5.4**	**1**	**R.VTGIPTPVVK.F** 1808
	1982	516.2933	1030.5720	1029.6070	0.9650	1	36	0.92	3	K.KALISEEIK.T
	2029	**518.8493**	**1035.6840**	**1035.6805**	**0.0035**	**0**	**41**	**0.0085**	**1**	**K.IVPGVIGLLR.A**
	2215	**528.2985**	**1054.5824**	**1054.5699**	**0.0125**	**0**	**15**	**55**	**3**	**R.YTVINLFGK.T**
	2259	**530.7965**	**1059.5784**	**1059.5672**	**0.0112**	**0**	**17**	**72**	**1**	**R.NALGSASATIR.L**
	2381	**358.8585**	**1073.5537**	**1073.5506**	**0.0032**	**0**	**22**	**26**	**2**	**R.TASPHFTVSK.I**
	2475	**362.8587**	**1085.5544**	**1085.5618**	**-0.0073**	**1**	**26**	**7.1**	**1**	**K.KIEAHFDAR.S**

	2917	567.2932	1132.5719	1132.5724	-0.0005	1	14	1.4e+002	3	K.TSGKLNIEDR.E
	3033	572.8409	1143.6671	1143.6434	0.0237	1	25	6.5	2	R.VNKVPVTMTR.Y
	3373	588.3191	1174.6237	1174.6234	0.0003	0	38	0.65	1	R.FGISEPLTSPK.M
	3667	600.7955	1199.5765	1199.5822	-0.0058	0	29	2.5	1	R.LVTGSEYQFR.V
	3699	601.8071	1201.5997	1200.5734	1.0263	0	29	4.2	1	R.AENAAGISEPSR.A
	4156	620.3228	1238.6311	1238.6295	0.0016	0	39	0.37	1	R.LIEGNEYVFR.V
	4963	653.8483	1305.6820	1305.6776	0.0044	0	60	0.0033	1	K.TIVSTAQISETR.Q 4961
	5731	690.3697	1378.7248	1376.7088	2.0160	0	18	49	1	K.VNWYLNGQLIR.K
	5842	463.9435	1388.8088	1388.8027	0.0061	0	65	0.00033	1	R.IEPLEVALGHLAK.F
	6446	724.8849	1447.7552	1446.7501	1.0051	0	37	0.65	1	K.SAAVATVVAAVDMAR.V
	7409	779.4080	1556.8013	1556.7875	0.0139	0	44	0.11	1	R.VSDLLEGVPYYFR.V
	7977	824.9211	1647.8277	1647.8620	-0.0343	1	18	51	2	K.DTASPILGYWLEKR.E
	8662	602.3106	1803.9100	1803.9115	-0.0015	0	30	3	1	R.TTTTAVHIQPAHEQIR.K
	8733	611.0059	1829.9958	1829.9927	0.0031	1	19	25	1	R.LLERPPEFTLPLYNK.T
6	gi|2959452 Mass: 52587 Score: 260 Sequences: 16 emPAI: 0.37<br>desmin [Bos taurus]									
	Query	Observed	Mr(expt)	Mr(calc)	Delta	Miss	Score	Expect	Rank	Peptide
	281	395.2216	788.4286	787.4075	1.0211	0	32	3.3	1	R.LEEEIR.H
	882	443.2239	884.4332	884.4279	0.0053	0	26	4.8	1	R.FANYIEK.V
	1694	498.2660	994.5174	994.4971	0.0204	0	33	4.2	1	R.AQYETIAAK.N
	1749	501.7656	1001.5165	1001.5142	0.0024	0	74	0.00012	1	R.ADVDAATLAR.I 1748
	1870	509.2781	1016.5417	1015.5411	1.0006	0	44	0.33	1	R.TSGGAGGLGALR.A 1860
	2495	544.7748	1087.5351	1086.5669	0.9682	0	21	25	1	R.QVEVLTNQR.A
	2805	561.7856	1121.5566	1121.5604	-0.0038	0	(20)	34	1	R.EYQDLLNVK.M
	2812	562.2849	1122.5553	1122.5444	0.0109	0	30	3.7	1	R.EYQDLLNVK.M

	4273	625.8176	1249.6207	1249.6190	0.0017	0	58	0.0061	1	R.VAEIYEEELR.E 4272
	4868	649.3288	1296.6431	1296.6384	0.0047	0	45	0.1	1	K.MALDVEIATYR.K
	6006	703.8798	1405.7451	1405.7340	0.0111	0	32	2	1	R.IESLNEEIAFLK.K
	7161	765.8492	1529.6839	1529.6634	0.0205	0	107	3.1e-008	1	R.FASEASGYQDNIAR.L
	7446	781.9264	1561.8382	1561.8351	0.0031	1	(27)	5.4	1	R.RIESLNEEIAFLK.K
	7447	521.6208	1561.8405	1561.8351	0.0054	1	51	0.022	1	R.RIESLNEEIAFLK.K
	7903	818.4279	1634.8412	1634.8668	-0.0256	0	44	0.31	1	R.INLPIQTFSALNFR.E 7908
	8125	838.4107	1674.8068	1674.8213	-0.0144	0	(30)	3.2	1	R.FLEQQNAALAAEVNR.L
	8135	838.9117	1675.8089	1675.8053	0.0036	0	(59)	0.0039	1	R.FLEQQNAALAAEVNR.L 8133
	8137	838.9214	1675.8282	1674.8213	1.0070	0	(23)	15	1	R.FLEQQNAALAAEVNR.L
	8141	839.4099	1676.8052	1676.7893	0.0160	0	103	1.6e-007	1	R.FLEQQNAALAAEVNR.L 8149
	8236	851.4224	1700.8301	1700.8217	0.0085	1	46	0.076	1	R.VDVERDNLLDDLQR.L
	8237	567.9511	1700.8316	1700.8217	0.0099	1	(24)	12	1	R.VDVERDNLLDDLQR.L
	9377	1059.5595	2117.1043	2117.0946	0.0097	0	95	7.2e-007	1	R.TFGGAPSFPLGSPLSSPVFPR.A 9376
	9378	706.7095	2117.1068	2117.0946	0.0122	0	(61)	0.0019	1	R.TFGGAPSFPLGSPLSSPVFPR.A
7	gi|115495853 Mass: 140689 Score: 114 Sequences: 19 emPAI: 0.06<br>myosin-binding protein C, cardiac-type [Bos taurus]									
	Query	Observed	Mr(expt)	Mr(calc)	Delta	Miss	Score	Expect	Rank	Peptide
	134	373.7152	745.4158	745.4123	0.0035	0	42	0.32	1	K.IDFVPR.Q
	447	412.7544	823.4942	822.4963	0.9979	0	35	0.33	1	K.EPVLIPR.P
	924	445.7595	889.5044	889.5021	0.0023	0	53	0.019	1	R.LNFDLLR.E
	926	446.2512	890.4879	890.4861	0.0017	0	(37)	0.7	1	R.LNFDLLR.E 925 927 928
	1119	460.7426	919.4706	918.4633	1.0073	0	23	18	1	K.LHFMEVK.I
	1237	470.2691	938.5237	938.5225	0.0012	0	30	1.9	1	K.VYLFELR.I
	1459	484.2495	966.4844	966.4770	0.0074	0	47	0.18	1	K.YSLAAEGTR.H

	1531	489.2454	976.4763	976.4648	0.0115	0	34	1.4	1	K.LLCETEGR.V
	2404	539.2610	1076.5075	1076.4774	0.0301	0	44	0.11	1	R.QAPPSEYER.I
	2687	554.7877	1107.5608	1107.5560	0.0048	0	45	0.1	1	R.SIFTVEGAER.E
	3484	592.2932	1182.5718	1182.5703	0.0015	0	28	4.4	1	R.VYAVNAVGMSR.P
	4355	629.3368	1256.6591	1256.6513	0.0077	0	(23)	35	1	R.IAFQHGVTDLR.G
	525	420.2320	1257.6741	1256.6513	1.0228	0	41	0.66	1	R.IAFQHGVTDLR.G 4357
	4553	636.8079	1271.6012	1271.5890	0.0123	0	51	0.037	1	R.MIEGVIYEMR.V
	4601	638.8451	1275.6757	1275.6823	-0.0065	0	16	94	1	R.NSPTDTILFIR.A
	4650	639.9118	1277.8090	1277.8071	0.0019	1	36	0.037	1	R.VAGASLLKPPVVK.W
	5256	667.3669	1332.7193	1332.7150	0.0043	0	(83)	1.6e-005	1	R.AHNLAGAGPPVTTK.E
	5265	667.8598	1333.7051	1333.6990	0.0061	0	89	4.2e-006	1	R.AHNLAGAGPPVTTK.E 5264
	6085	707.8391	1413.6637	1413.6525	0.0112	0	32	1.3	1	R.AGAGGLDGYSVEYR.R
	6418	724.3317	1446.6488	1446.6449	0.0039	0	42	0.1	1	R.IMDAQTTFAGGYR.C 6433
	7476	522.9572	1565.8497	1565.8413	0.0084	1	56	0.0057	1	K.EPVTVQELLQRPR.L 2096
	7855	543.2578	1626.7516	1626.7501	0.0016	0	46	0.066	1	K.TMEWFTVLEHYR.H
**8**	**gi|1351907 Mass: 71244 Score: 545 Sequences: 21 emPAI: 1.13**<br>**RecName: Full=Serum albumin; AltName: Full=BSA; AltName: Allergen=Bos d 6; Flags: Precursor**									
	**Query**	**Observed**	**Mr(expt)**	**Mr(calc)**	**Delta**	**Miss**	**Score**	**Expect**	**Rank**	**Peptide**
	287	395.2403	788.4660	788.4644	0.0017	0	26	8.9	1	K.LVTDLTK.V 285
	628	424.7477	847.4809	847.4803	0.0006	1	23	18	3	R.LSQKFPK.A 629
	1154	464.2516	926.4886	926.4861	0.0024	0	41	0.18	1	K.YLYEIAR.R 1155 1156 1157
	1846	507.8140	1013.6134	1013.6121	0.0014	0	(37)	0.31	1	K.QTALVELLK.H 1856
	1855	508.3045	1014.5944	1014.5961	-0.0017	0	67	0.00044	1	K.QTALVELLK.H
	2674	554.2662	1106.5179	1106.5066	0.0112	0	52	0.019	1	K.EACFAVEGPK.L
	2970	570.2506	1138.4866	1138.4747	0.0119	0	25	9.5	1	K.CCTESLVNR.R

	3228	582.3202	1162.6259	1162.6234	0.0026	0	74	0.00013	1	K.LVNELTEFAK.T
	3250	582.8127	1163.6108	1163.6074	0.0034	0	(58)	0.0051	1	K.LVNELTEFAK.T 3247 3251
	4262	417.2210	1248.6412	1248.6139	0.0274	1	32	2.4	1	R.FKDLGEEHFK.G
	4701	642.3591	1282.7037	1282.7034	0.0003	0	26	4.9	1	R.HPEYAVSVLLR.L
	6101	708.3512	1414.6879	1414.6803	0.0076	0	(67)	0.00051	1	K.TVMENFVAFVDK.C 6103
	6109	708.8405	1415.6664	1415.6643	0.0021	0	85	6.8e-006	1	K.TVMENFVAFVDK.C
	6147	710.3536	1418.6926	1418.6864	0.0062	0	29	4.3	1	K.SLHTLFGDELCK.V
	6377	480.9437	1439.8093	1438.8045	1.0048	1	74	6.6e-005	1	R.RHPEYAVSVLLR.L 1401 1402 6367
	6553	488.5371	1462.5894	1462.5817	0.0077	0	74	1.9e-005	1	K.TCVADESHAGCEK.S
	6723	740.3945	1478.7744	1478.7881	-0.0137	0	(98)	5.3e-007	1	K.LGEYGFQNALIVR.Y 6722
	6754	740.8960	1479.7775	1479.7722	0.0053	0	113	1.6e-008	1	K.LGEYGFQNALIVR.Y
	6755	740.8967	1479.7788	1479.7722	0.0067	0	(47)	0.059	1	K.LGEYGFQNALIVR.Y
	6757	741.3857	1480.7568	1480.7562	0.0006	0	(105)	2.4e-007	1	K.LGEYGFQNALIVR.Y 6758 6769 6770
	7029	756.4107	1510.8068	1510.8355	-0.0287	0	(44)	0.098	1	K.VPQVSTPTLVEVSR.S
	7038	756.9149	1511.8151	1511.8195	-0.0044	0	47	0.042	1	K.VPQVSTPTLVEVSR.S
	7477	784.3775	1566.7405	1566.7354	0.0051	0	75	8.9e-005	1	K.DAFLGSFLYEYSR.R 7478 7479 7480 7481 7486
	7926	820.4736	1638.9327	1638.9305	0.0022	1	(66)	0.00024	1	R.KVPQVSTPTLVEVSR.S 7925 7927 7928
	7931	547.3187	1638.9342	1638.9305	0.0037	1	(58)	0.0013	1	R.KVPQVSTPTLVEVSR.S 7929 7930
	7939	547.6469	1639.9190	1639.9145	0.0045	1	83	7.1e-006	1	R.KVPQVSTPTLVEVSR.S 7944
	8405	871.4147	1740.8149	1740.8062	0.0087	0	122	1.6e-009	1	R.MPCTEDYLSLILNR.L 8406 8407
	8883	940.9682	1879.9219	1879.9138	0.0081	1	51	0.023	1	R.RPCFSALTPDETYVPK.A
	8918	945.4610	1888.9074	1888.9035	0.0038	0	21	56	1	R.HPYFYAPELLYYANK.Y
	9251	682.3490	2044.0253	2044.0206	0.0046	1	(14)	1.3e+002	1	R.RHPYFYAPELLYYANK.Y
	9255	683.0141	2046.0203	2045.0046	1.0157	1	25	11	1	R.RHPYFYAPELLYYANK.Y 5558

	Query	Observed	Mr(expt)	Mr(calc)	Delta	Miss	Score	Expect	Rank	Peptide
**9**	**gi|27806939 Mass: 17067 Score: 358 Sequences: 6 emPAI: 1.05** **myoglobin [Bos taurus]**									
	**Query**	**Observed**	**Mr(expt)**	**Mr(calc)**	**Delta**	**Miss**	**Score**	**Expect**	**Rank**	**Peptide**
	141	**374.7248**	**747.4350**	**747.4279**	**0.0071**	**0**	**37**	**0.67**	**1**	**K.ALELFR.N** 142 143 144
	4544	**636.3364**	**1270.6583**	**1270.6557**	**0.0026**	**0**	**62**	**0.0017**	**1**	**R.LFTGHPETLEK.F** 4545
	5875	**697.4061**	**1392.7976**	**1392.8089**	**-0.0113**	**0**	**(57)**	**0.0022**	**1**	**K.HGNTVLTALGGILK.K**
	5888	**697.9028**	**1393.7911**	**1393.7929**	**-0.0018**	**0**	**94**	**4.8e-007**	**1**	**K.HGNTVLTALGGILK.K** 5887 5890 5891
	7339	**517.2273**	**1548.6601**	**1548.6515**	**0.0086**	**0**	**(27)**	**1.8**	**1**	**K.HPSDFGADAQAAMSK.A**
	7340	**775.3384**	**1548.6623**	**1548.6515**	**0.0108**	**0**	**47**	**0.019**	**1**	**K.HPSDFGADAQAAMSK.A** 7341
	7656	**797.4162**	**1592.8179**	**1592.8158**	**0.0021**	**0**	**(51)**	**0.024**	**1**	**K.VEADVAGHGQEVLIR.L**
	7660	**531.9493**	**1592.8262**	**1592.8158**	**0.0104**	**0**	**124**	**1.2e-009**	**1**	**K.VEADVAGHGQEVLIR.L** 7657 7658 7659 7661 7662 2276 2278
	8845	**623.6820**	**1868.0241**	**1868.0196**	**0.0045**	**0**	**127**	**3e-010**	**1**	**K.YLEFISDAIIHVLHAK.H**
**10**	**gi|6006425 Mass: 15201 Score: 87 Sequences: 4 emPAI: 0.72** **hemoglobin alpha chain [Bos taurus]**									
	**Query**	**Observed**	**Mr(expt)**	**Mr(calc)**	**Delta**	**Miss**	**Score**	**Expect**	**Rank**	**Peptide**
	423	**410.2173**	**818.4200**	**818.4174**	**0.0026**	**0**	**48**	**2.8**	**1**	**R.VDPVNFK.L** 422
	2483	**544.2801**	**1086.5456**	**1086.5420**	**0.0037**	**0**	**28**	**4.1**	**1**	**R.MFLSFPTTK.T** 2482
	4672	**640.8645**	**1279.7145**	**1279.7024**	**0.0121**	**0**	**66**	**0.00053**	**1**	**K.FLANVSTVLTSK.Y** 4671
	7164	**510.9222**	**1529.7447**	**1528.7270**	**1.0178**	**0**	**82**	**1.7e-005**	**1**	**K.VGGHAAEYGAEALER.M**
**11**	**gi|219804724 Mass: 109744 Score: 55 Sequences: 10** **collagen alpha-1(VI) chain precursor [Bos taurus]**									
	**Query**	**Observed**	**Mr(expt)**	**Mr(calc)**	**Delta**	**Miss**	**Score**	**Expect**	**Rank**	**Peptide**
	349	402.2210	802.4275	802.3821	0.0455	0	20	41	7	K.AVQEAQR.A
	1384	479.2903	956.5661	956.5655	0.0007	0	54	0.0081	1	R.IALVITDGR.S

	1796	**504.7648**	**1007.5150**	**1006.4720**	**1.0430**	**0**	**20**	**37**	**1**	**R.FIDNLNDR.Y**
	1923	**512.2728**	**1022.5310**	**1022.5396**	**-0.0086**	**0**	**29**	**3.6**	**1**	**R.LLPPTPNNR.I** 1922
	2137	523.8024	1045.5902	1045.5920	-0.0018	0	(40)	0.32	1	R.VPSYQALLR.G
	2142	524.2977	1046.5808	1046.5760	0.0048	0	63	0.0018	1	R.VPSYQALLR.G 2141 2143
	2225	529.2730	1056.5313	1056.5240	0.0073	0	17	44	1	R.GVFYQTVSR.K
	2638	552.3192	1102.6239	1102.6386	-0.0148	1	56	0.0071	1	R.LKPYGALVDK.V
	3857	**608.8542**	**1215.6937**	**1215.6976**	**-0.0038**	**0**	**36**	**0.61**	**1**	**R.AGVEIFAVVVGR.Q** 3858 3874 3876
	4323	**628.3030**	**1254.5915**	**1254.5881**	**0.0035**	**0**	**39**	**0.26**	**1**	**K.AAEYDVVFGER.H** 4324
	4777	645.3494	1288.6842	1288.6775	0.0067	0	37	1.4	1	R.LSIIATDHTYR.R 4786
**12**	**gi|297473452 Mass: 277630 Score: 53 Sequences: 24<br>PREDICTED: collagen alpha-3(VI) chain isoform 4 [Bos taurus]**									
	**Query**	**Observed**	**Mr(expt)**	**Mr(calc)**	**Delta**	**Miss**	**Score**	**Expect**	**Rank**	**Peptide**
	27	**358.7149**	**715.4153**	**715.4228**	**-0.0076**	**0**	**39**	**0.42**	**1**	**K.VDTILR.R**
	73	**363.6856**	**725.3567**	**725.3530**	**0.0036**	**0**	**20**	**19**	**1**	**R.SGFLMR.K** 72
	80	**364.7244**	**727.4341**	**726.4388**	**0.9953**	**0**	**43**	**0.082**	**1**	**R.GPSVVLR.R**
	100	**367.7381**	**733.4617**	**733.4487**	**0.0130**	**0**	**28**	**2**	**1**	**R.LVFTVR.E**
	138	**374.2303**	**746.4459**	**746.4439**	**0.0020**	**0**	**37**	**0.76**	**1**	**K.VFAVGVR.N**
	183	**381.2322**	**760.4498**	**760.4483**	**0.0014**	**0**	**47**	**0.07**	**1**	**R.SGFPLLK.E**
	345	**401.7382**	**801.4617**	**800.4392**	**1.0226**	**0**	**24**	**16**	**1**	**R.EVINAVR.K**
	356	**403.2332**	**804.4519**	**804.4494**	**0.0025**	**0**	**36**	**0.86**	**3**	**K.ALEFVAR.N**
	1391	**479.7482**	**957.4819**	**957.4767**	**0.0052**	**0**	**18**	**45**	**2**	**R.DLPSIEER.M**
	1500	**487.2756**	**972.5367**	**972.5352**	**0.0015**	**0**	**18**	**60**	**3**	**R.NILVGSAGSR.I**
	1589	**492.3185**	**982.6224**	**982.6175**	**0.0049**	**0**	**45**	**0.015**	**1**	**R.ALILVGLER.V** 1588 1590
	2025	**518.7715**	**1035.5284**	**1035.5349**	**-0.0065**	**0**	**41**	**0.26**	**1**	**R.VAVVQYSDR.T**
	2039	**519.7709**	**1037.5273**	**1036.5189**	**1.0084**	**0**	**(37)**	**0.65**	**1**	**R.VAVVQYSDR.T** 2040

	2254	530.7855	1059.5565	1058.5720	0.9845	0	22	29	1	K.SQASVLDAIR.R 2256
	2257	530.7889	1059.5633	1059.5560	0.0073	0	(15)	1.9e+002	1	K.SQASVLDAIR.R
	3349	587.3346	1172.6545	1172.6554	-0.0008	0	24	12	1	K.QFGVAPLTIAR.N
	3501	593.3100	1184.6055	1184.6037	0.0017	0	22	16	1	R.VVESLDVGPDR.V
	4054	615.8516	1229.6887	1229.7020	-0.0133	0	31	2	1	R.VPQIAFVITGGK.S
	4456	633.3305	1264.6464	1264.6928	-0.0464	1	27	6.6	1	K.VAVFFSNKPTR.A
	4534	424.2169	1269.6287	1269.6214	0.0073	1	30	2.6	1	R.RNHFVPEAGSR.L
	5676	687.8646	1373.7147	1373.7038	0.0110	0	27	6.6	1	R.QLGTIQQVISER.V
	6623	734.8749	1467.7351	1467.7357	-0.0006	0	26	7.8	1	K.ALNLGYALDYAQR.Y 6633
	6786	743.3533	1484.6921	1484.6817	0.0104	0	41	0.19	1	K.QQSLETAMSFVAR.N
	8069	833.3916	1664.7687	1664.8079	-0.0393	0	22	43	1	R.VANLEQLMQLEFGR.G
	8278	855.4454	1708.8762	1708.8519	0.0243	0	32	3.7	1	R.VAVVTYNNEVTTEIR.F
	8820	932.4743	1862.9340	1862.9262	0.0079	0	53	0.017	1	K.LSDAGITPLFLTSQEDR.Q

Table S2 Details of peptides of stick2 (from M22) identified by searching against the NCBInr database

<table>
<tr><td>1</td><td colspan="10">gi|41386711 Mass: 223889 Score: 433 Sequences: 35 emPAI: 0.23
myosin-7 [Bos taurus]</td></tr>
<tr><td></td><td>Query</td><td>Observed</td><td>Mr(expt)</td><td>Mr(calc)</td><td>Delta</td><td>Miss</td><td>Score</td><td>Expect</td><td>Rank</td><td>Peptide</td></tr>
<tr><td></td><td>117</td><td>374.2418</td><td>746.4689</td><td>746.4691</td><td>-0.0001</td><td>0</td><td>(18)</td><td>36</td><td>4</td><td>R.VIFQLK.A</td></tr>
<tr><td></td><td>122</td><td>374.7338</td><td>747.4530</td><td>747.4531</td><td>-0.0000</td><td>0</td><td>30</td><td>2.5</td><td>1</td><td>R.VIFQLK.A 121</td></tr>
<tr><td></td><td>183</td><td>384.2308</td><td>766.4470</td><td>766.4490</td><td>-0.0019</td><td>1</td><td>28</td><td>2.3</td><td>1</td><td>R.FGKFIR.I</td></tr>
<tr><td></td><td>254</td><td>395.2416</td><td>788.4685</td><td>788.4643</td><td>0.0042</td><td>0</td><td>45</td><td>0.17</td><td>1</td><td>K.SLQSLLK.D</td></tr>
</table>

	444	415.7284	829.4423	829.4446	-0.0023	0	20	35	1	R.IHFGATGK.L
	813	442.2371	882.4596	882.4599	-0.0004	0	45	0.069	1	R.ILYGDFR.Q 812 814
	854	445.2533	888.4920	888.4916	0.0004	0	(13)	3.8e+002	1	R.INATLETK.Q
	860	445.7449	889.4753	889.4756	-0.0003	0	15	1.3e+002	1	R.INATLETK.Q
	1093	462.7553	923.4960	923.4964	-0.0004	0	67	0.00051	1	R.SLSTELFK.L 1092
	1555	494.2655	986.5165	985.5556	0.9609	1	14	1.3e+002	2	K.QLQKLEAR.V
	1586	496.2919	990.5693	990.5862	-0.0169	1	26	6.3	1	K.SRVIFQLK.A
	1701	502.7497	1003.4848	1003.4822	0.0027	0	27	5.9	1	R.ELEEISER.L
	1821	511.2453	1020.4760	1020.4724	0.0037	0	33	1.3	1	R.SVNDLTSQR.A 1824
	1913	516.2899	1030.5653	1030.5658	-0.0005	0	50	0.034	1	K.EALISQLTR.G
	2182	531.2552	1060.4959	1060.4937	0.0021	0	43	0.15	1	K.ANSEVAQWR.T
	79	368.8871	1103.6394	1103.6339	0.0056	1	56	0.0052	1	R.VIFQLKAER.D
	2664	558.7503	1115.4859	1115.4892	-0.0033	0	(36)	0.25	1	R.MFNWMVTR.I 2663
	2668	559.2436	1116.4726	1116.4732	-0.0006	0	39	0.11	1	R.MFNWMVTR.I
	3150	389.8798	1166.6177	1166.6196	-0.0020	1	(20)	25	1	R.ILYGDFRQR.Y
	3163	584.8086	1167.6026	1167.6036	-0.0010	1	39	0.38	1	R.ILYGDFRQR.Y
	3166	390.2097	1167.6073	1167.6036	0.0037	1	(38)	0.41	1	R.ILYGDFRQR.Y 3164 3167 3181
	3364	396.2347	1185.6823	1185.6829	-0.0007	1	(26)	5.2	1	R.IITRIQAQSR.G
	3365	396.2347	1185.6823	1185.6829	-0.0007	1	(28)	3.3	1	R.IITRIQAQSR.G
	3377	594.3402	1186.6659	1186.6669	-0.0010	1	(35)	0.78	1	R.IITRIQAQSR.G
	3380	396.5639	1186.6697	1186.6669	0.0028	1	47	0.057	1	R.IITRIQAQSR.G 3379
	3713	409.2116	1224.6131	1224.5986	0.0145	1	32	1.6	1	R.TKYETDAIQR.T
	4181	424.2417	1269.7033	1269.7041	-0.0008	1	(88)	3.3e-006	1	R.INATLETKQPR.Q
	4190	636.3508	1270.6871	1270.6881	-0.0010	1	(65)	0.00088	1	R.INATLETKQPR.Q

	4191	424.5704	1270.6894	1270.6881	0.0013	1	(47)	0.05	1	R.INATLETKQPR.Q
	4192	424.5704	1270.6895	1270.6881	0.0014	1	(48)	0.037	1	R.INATLETKQPR.Q 4197
	4199	424.8986	1271.6740	1271.6721	0.0019	1	89	3.9e-006	1	R.INATLETKQPR.Q 4198 4201 4213
	4901	449.2384	1344.6932	1344.6885	0.0048	1	62	0.0021	1	R.ADIAESQVNKLR.A
	4914	674.3374	1346.6602	1346.6677	-0.0075	1	(15)	2.7e+002	1	R.AKLQTENGELSR.Q
	4916	449.8983	1346.6731	1345.6837	0.9894	1	44	0.15	1	R.AKLQTENGELSR.Q
	5090	455.9030	1364.6872	1362.6813	2.0059	1	18	47	1	R.ENLNKLMTNLR.S
	5543	709.8587	1417.7028	1416.7096	0.9932	0	28	5.8	1	K.LAEQELIETSER.V
	5903	733.3863	1464.7581	1464.7711	-0.0130	0	40	0.35	1	K.LASADIETYLLEK.S 5904 5910
	6034	495.2462	1482.7168	1482.6950	0.0218	1	61	0.0024	1	R.KVQHELDEAEER.A
	6073	744.8571	1487.6996	1487.6991	0.0006	0	80	2.6e-005	1	R.IEELEEELEAER.T 6072
	1732	504.2655	1509.7748	1508.7722	1.0026	1	49	0.12	1	K.LTYTQQLEDLKR.Q
	6361	767.8847	1533.7548	1533.7522	0.0026	0	61	0.0026	1	R.VVDSLQTSLDAETR.S 6362
	6369	768.4272	1534.8399	1534.8508	-0.0109	0	(52)	0.023	1	R.VIQYFAVIAAIGDR.S 6366 6367 6368 6372
	6374	768.9230	1535.8314	1535.8348	-0.0033	0	55	0.0071	1	R.VIQYFAVIAAIGDR.S 6373 6375 6376 6380
	6440	773.8995	1545.7844	1545.7886	-0.0042	1	80	3.4e-005	1	R.KLAEQELIETSER.V
	3104	582.2943	1743.8610	1743.8638	-0.0029	2	23	49	1	R.VRELENELEAEQKR.N
	7570	920.4576	1838.9006	1838.9010	-0.0004	0	130	3.1e-010	1	R.DLEEATLQHEATAAALR.K
	7571	613.9748	1838.9026	1838.9010	0.0016	0	(18)	49	1	R.DLEEATLQHEATAAALR.K
	7654	624.0065	1868.9976	1868.9996	-0.0020	1	39	0.24	1	R.ILNPAAIPEGQFIDSRK.G
	7845	656.3392	1965.9959	1966.0119	-0.0160	1	13	1.4e+002	1	R.DLEEATLQHEATAAALRK.K
	2768	564.8056	2255.1934	2255.1797	0.0137	2	13	2.3e+002	1	R.ILNPAAIPEGQFIDSRKGAEK.L
**2**	**gi|61888866 Mass: 32732 Score: 230 Sequences: 4 tropomyosin alpha-1 chain [Bos taurus]**									
	**Query**	**Observed**	**Mr(expt)**	**Mr(calc)**	**Delta**	**Miss**	**Score**	**Expect**	**Rank**	**Peptide**

	**387**	**593.8369**	**1185.6592**	**1185.6605**	**-1.04**	**0**	**48**	**0.51**	**1**	**K.LVIIESDLER.A**
	**467**	**622.8215**	**1243.6284**	**1243.6296**	**-0.90**	**0**	**82**	**0.0002**	**1**	**R.IQLVEEELDR.A**
	**614**	**657.8866**	**1313.7586**	**1313.7554**	**2.45**	**1**	**59**	**0.028**	**1**	**R.KLVIIESDLER.A**
	**664**	**667.3153**	**1332.6160**	**1332.6157**	**0.26**	**0**	**40**	**2.6**	**1**	**K.ATDAEADVASLNR.R**
**3**	**gi|296486595 Mass: 29302 Score: 102 Sequences: 4 emPAI: 0.32**<br>**tropomyosin 4-like [Bos taurus]**									
	**Query**	**Observed**	**Mr(expt)**	**Mr(calc)**	**Delta**	**Miss**	**Score**	**Expect**	**Rank**	**Peptide**
	4566	436.8827	1307.6261	1307.6106	0.0156	1	59	0.0041	1	K.EAETRAEFAER.S
	5975	738.3947	1474.7748	1474.7701	0.0047	1	15	1.1e+002	5	K.MELQEIQLKEAK.H
	7228	576.9662	1727.8767	1727.8689	0.0078	1	(15)	97	1	R.IQLVEEELNRAQER.L
	7233	865.4344	1728.8543	1728.8529	0.0014	1	(34)	1.3	1	R.IQLVEEELNRAQER.L
	7234	577.2941	1728.8604	1728.8529	0.0074	1	39	0.38	1	R.IQLVEEELNRAQER.L
	7685	628.9966	1883.9680	1883.9700	-0.0021	2	(44)	0.11	1	R.RIQLVEEELNRAQER.L
	7688	629.3249	1884.9529	1884.9540	-0.0011	2	87	5.4e-006	1	R.RIQLVEEELNRAQER.L
	7689	472.2476	1884.9613	1884.9540	0.0073	2	(57)	0.0053	1	R.RIQLVEEELNRAQER.L
**4**	**gi|297465038 Mass: 3741571 Score: 172 Sequences: 31 emPAI: 0.00**<br>**PREDICTED: LOW QUALITY PROTEIN: titin [Bos taurus]**									
	**Query**	**Observed**	**Mr(expt)**	**Mr(calc)**	**Delta**	**Miss**	**Score**	**Expect**	**Rank**	**Peptide**
	4	350.7215	699.4284	699.4279	0.0005	0	21	14	1	R.LIGELR.L
	30	358.7306	715.4467	715.4381	0.0086	0	17	51	1	R.FPAIIR.G
	33	358.7367	715.4589	715.4632	-0.0043	0	22	13	1	R.IFVPIK.G
	38	359.7344	717.4541	717.4537	0.0004	0	30	2.1	1	R.LFAIVR.G
	202	387.2278	772.4410	772.4443	-0.0033	0	20	36	1	R.ISTSPIR.S
	263	397.2158	792.4169	792.4130	0.0040	0	13	1.7e+002	1	K.SAFVNVR.V
	269	398.2339	794.4532	794.4538	-0.0006	0	16	54	4	K.SITFTVK.V

	313	403.2490	804.4835	804.4858	-0.0023	0	33	1.1	1	R.LFVTGIR.L
	355	407.2591	812.5037	812.5120	-0.0083	0	35	0.33	1	K.LIIEGIR.L
	580	426.2191	850.4236	850.4218	0.0017	0	18	45	8	R.LQSMTVR.Q
	687	**433.7218**	**865.4291**	**865.4294**	**-0.0002**	**0**	**19**	**41**	**6**	**K.AYANVSNK.C** 685
	892	448.3009	894.5873	894.5902	-0.0029	0	26	0.53	1	R.VLGVPVIAK.D
	1020	458.7596	915.5046	915.5389	-0.0343	0	14	1.6e+002	6	K.ITLVDVTR.N
	1375	**481.7516**	**961.4886**	**961.5154**	**-0.0268**	**0**	**16**	**1.1e+002**	**3**	**R.LELSQMLK.K**
	1751	505.8114	1009.6083	1009.6172	-0.0089	0	26	2.2	1	R.VTGIPTPVVK.F
	1820	510.7778	1019.5411	1019.5400	0.0011	0	52	0.026	1	R.VGEAFALTGR.Y 1819
	1913	516.2899	1030.5653	1029.6070	0.9584	1	34	1.5	3	K.KALISEEIK.T
	2075	**524.7673**	**1047.5201**	**1046.5244**	**0.9957**	**0**	**19**	**45**	**1**	**R.NGVVLESSDK.Y**
	18	355.2125	1062.6156	1062.6073	0.0083	1	31	4.1	1	R.LIKEIEYR.I
	2413	363.1959	1086.5658	1085.5618	1.0040	1	28	5.6	1	K.KIEAHFDAR.S
	63	365.2179	1092.6320	1091.6564	0.9757	1	23	24	1	K.ALVQGRPVPR.V
	2483	365.2214	1092.6424	1092.6404	0.0020	1	(18)	21	1	K.ALVQGRPVPR.V
	2570	368.8887	1103.6442	1103.6451	-0.0010	1	18	28	1	K.IIGYVVERR.D
	2837	379.2208	1134.6406	1134.6397	0.0009	1	50	0.02	1	R.ITNYIVEKR.E
	2845	379.5505	1135.6296	1135.6237	0.0060	1	(48)	0.04	1	R.ITNYIVEKR.E
	3238	588.8240	1175.6334	1174.6234	1.0100	0	30	3.1	1	R.FGISEPLTSPK.M
	3513	601.7972	1201.5798	1200.5734	1.0063	0	68	0.00037	1	R.AENAAGISEPSR.A
	4005	627.3265	1252.6383	1252.6452	-0.0068	0	60	0.0026	1	R.LIEGNEYIFR.V
	4008	418.5684	1252.6834	1252.6815	0.0018	1	73	0.0001	1	R.LIEGVKYQFR.A 4019
	778	439.2502	1314.7288	1314.7296	-0.0008	1	79	8.7e-005	1	K.VGLKGVEFNVPR.L
	5219	461.2578	1380.7515	1380.7612	-0.0098	1	25	6.3	1	R.VLAENLAGPGKPSK.S

	5814	486.2530	1455.7372	1455.7430	-0.0057	1	54	0.013	1	R.VRAENAAGISEPSR.A
	2726	562.2930	1683.8572	1683.8791	-0.0219	1	47	0.19	1	R.VREPVISAVEQTAQR.T
**5**	**gi|2959452 Mass: 52587 Score: 302 Sequences: 5 emPAI: 0.07**<br>**desmin [Bos taurus]**									
	**Query**	**Observed**	**Mr(expt)**	**Mr(calc)**	**Delta**	**Miss**	**Score**	**Expect**	**Rank**	**Peptide**
	479	625.8173	1249.6200	1249.6190	0.85	0	64	0.017	1	R.VAEIYEEELR.E
	564	649.3261	1296.6376	1296.6384	-0.55	0	56	0.099	1	K.MALDVEIATYR.K
	987	765.8381	1529.6616	1529.6634	-1.13	0	34	4.5	1	R.FASEASGYQDNIAR.L
	1029	521.6184	1561.8334	1561.8351	-1.12	1	(38)	7	1	R.RIESLNEEIAFLK.K
	1030	781.9257	1561.8368	1561.8351	1.11	1	54	0.17	1	R.RIESLNEEIAFLK.K
	1185	838.9075	1675.8004	1675.8053	-2.88	0	94	1.6e-005	1	R.FLEQQNAALAAEVNR.L
	1186	839.4011	1676.7876	1676.7893	-0.97	0	(58)	0.068	1	R.FLEQQNAALAAEVNR.L
**6**	**gi|115495853 Mass: 140689 Score: 70 Sequences: 5 emPAI: 0.03**<br>**myosin-binding protein C, cardiac-type [Bos taurus]**									
	**Query**	**Observed**	**Mr(expt)**	**Mr(calc)**	**Delta**	**Miss**	**Score**	**Expect**	**Rank**	**Peptide**
	1889	514.8063	1027.5980	1027.6026	-0.0046	0	23	9.2	1	K.QGVLTLEIR.K 1894
	2332	539.7510	1077.4875	1075.4934	1.9941	0	26	5.1	1	R.QAPPSEYER.I 2323
	3110	582.3056	1162.5966	1162.6234	-0.0268	1	20	44	1	K.KLQPAYQVSK.G
	3327	395.2234	1182.6483	1182.6397	0.0086	1	51	0.016	1	K.YIFESIGAKR.T
	4273	426.9441	1277.8106	1277.8071	0.0035	1	53	0.00075	1	R.VAGASLLKPPVVK.W
**7**	**gi|1351907 Mass: 71244 Score: 1191 Sequences: 21 emPAI: 0.11**<br>**RecName: Full=Serum albumin; AltName: Full=BSA; AltName: Allergen=Bos d 6; Flags: Precursor**									
	**Query**	**Observed**	**Mr(expt)**	**Mr(calc)**	**Delta**	**Miss**	**Score**	**Expect**	**Rank**	**Peptide**
	37	**395.2391**	**788.4636**	**788.4644**	**-0.92**	**0**	**49**	**0.5**	**1**	**K.LVTDLTK.V**
	71	**424.2548**	**846.4950**	**846.4963**	**-1.51**	**1**	**28**	**39**	**1**	**R.LSQKFPK.A**

	118	464.2501	926.4856	926.4861	-0.52	0	30	17	5	K.YLYEIAR.R
	178	501.7949	1001.5752	1001.5757	-0.47	0	17	6.7e+002	3	K.LVVSTQTALA.-
	188	507.8128	1013.6110	1013.6121	-1.01	0	66	0.0039	1	K.QTALVELLK.H
	320	571.8607	1141.7068	1141.7070	-0.16	1	75	0.00026	1	K.KQTALVELLK.H
	321	381.5764	1141.7074	1141.7070	0.30	1	(46)	0.2	1	K.KQTALVELLK.H
	350	582.3191	1162.6236	1162.6234	0.24	0	63	0.017	1	K.LVNELTEFAK.T
	476	417.2119	1248.6139	1248.6139	-0.00	1	(46)	1.1	1	R.FKDLGEEHFK.G
	477	625.3157	1248.6168	1248.6139	2.38	1	57	0.09	1	R.FKDLGEEHFK.G
	539	642.3584	1282.7022	1282.7034	-0.86	0	57	0.046	1	R.HPEYAVSVLLR.L
	592	435.9102	1304.7088	1304.7088	-0.05	0	(43)	2	1	K.HLVDEPQNLIK.Q
	593	653.3618	1304.7090	1304.7088	0.17	0	57	0.085	1	K.HLVDEPQNLIK.Q
	847	480.6089	1438.8049	1438.8045	0.29	1	68	0.0035	1	R.RHPEYAVSVLLR.L
	848	720.4101	1438.8056	1438.8045	0.83	1	(47)	0.38	1	R.RHPEYAVSVLLR.L
	854	722.3281	1442.6416	1442.6347	4.78	0	24	69	1	K.YICDNQDTISSK.L
	909	493.9366	1478.7880	1478.7881	-0.12	0	(49)	0.48	1	K.LGEYGFQNALIVR.Y
	910	740.4020	1478.7894	1478.7881	0.88	0	93	1.9e-005	1	K.LGEYGFQNALIVR.Y 907 908 911 914 915
	956	504.6187	1510.8343	1510.8355	-0.83	0	(28)	35	1	K.VPQVSTPTLVEVSR.S
	957	756.4259	1510.8372	1510.8355	1.14	0	76	0.00064	1	K.VPQVSTPTLVEVSR.S
	959	756.9165	1511.8184	1511.8195	-0.72	0	(71)	0.0026	2	K.VPQVSTPTLVEVSR.S
	1012	516.3033	1545.8881	1545.8878	0.14	1	50	0.16	1	K.LKHLVDEPQNLIK.Q
	1049	784.3749	1566.7352	1566.7354	-0.12	0	79	0.00047	1	K.DAFLGSFLYEYSR.R 1045 1047 1048 1051 1052 1055 1056 1057
	1158	820.4732	1638.9318	1638.9305	0.83	1	(78)	0.00023	1	R.KVPQVSTPTLVEVSR.S
	1159	547.6464	1639.9174	1639.9145	1.76	1	85	6.4e-005	1	R.KVPQVSTPTLVEVSR.S
	1160	820.9669	1639.9192	1639.9145	2.90	1	(72)	0.0014	1	R.KVPQVSTPTLVEVSR.S

	1161	**547.6523**	**1639.9351**	**1638.9305**	**613**	**1**	**(51)**	**0.12**	**1**	**R.KVPQVSTPTLVEVSR.S**
	1406	**940.9645**	**1879.9144**	**1879.9138**	**0.33**	**1**	**32**	**34**	**1**	**R.RPCFSALTPDETYVPK.A**
	1407	**627.6457**	**1879.9153**	**1879.9138**	**0.76**	**1**	**(16)**	**1.4e+003**	**1**	**R.RPCFSALTPDETYVPK.A**
	1410	**630.3135**	**1887.9187**	**1887.9195**	**-0.45**	**0**	**(40)**	**5.1**	**1**	**R.HPYFYAPELLYYANK.Y**
	1411	**944.9669**	**1887.9192**	**1887.9195**	**-0.15**	**0**	**72**	**0.0031**	**1**	**R.HPYFYAPELLYYANK.Y**
	1484	**978.4832**	**1954.9518**	**1954.9524**	**-0.26**	**0**	**64**	**0.02**	**1**	**K.DAIPENLPPLTADFAEDK.D**
	1547	**682.3474**	**2044.0204**	**2044.0206**	**-0.13**	**1**	**50**	**0.62**	**1**	**R.RHPYFYAPELLYYANK.Y**
	1548	**512.0131**	**2044.0233**	**2044.0206**	**1.30**	**1**	**(27)**	**1.3e+002**	**1**	**R.RHPYFYAPELLYYANK.Y**
	1549	**1023.0208**	**2044.0270**	**2044.0206**	**3.14**	**1**	**(38)**	**10**	**1**	**R.RHPYFYAPELLYYANK.Y**
**8**	**gi|122286 Mass: 14972 Score: 56 Sequences: 5 emPAI: 0.31** **RecName: Full=Hemoglobin subunit alpha-2; AltName: Full=Alpha-2-globin; AltName: Full=Hemoglobin alpha-2 chain**									
	**Query**	**Observed**	**Mr(expt)**	**Mr(calc)**	**Delta**	**Miss**	**Score**	**Expect**	**Rank**	**Peptide**
	387	409.7236	817.4327	817.4334	-0.0007	0	(34)	1.9	1	R.VDPVNFK.L 386
	391	410.2164	818.4183	818.4174	0.0009	0	53	0.021	1	R.VDPVNFK.L 390 392
	2410	**544.2772**	**1086.5399**	**1086.5420**	**-0.0021**	**0**	**46**	**0.071**	**1**	**R.MFLSFPTTK.T** 2415
	2417	544.3125	1086.6103	1086.6186	-0.0082	1	(18)	52	1	K.LRVDPVNFK.L
	2418	363.2145	1086.6217	1086.6186	0.0031	1	41	0.22	1	K.LRVDPVNFK.L
	2441	363.8790	1088.6152	1087.6026	1.0126	1	(38)	0.53	1	K.LRVDPVNFK.L
	6329	510.5835	1528.7287	1528.7270	0.0017	0	61	0.0022	3	K.VGGHAAEYGAEALER.M 6336
	6330	765.3731	1528.7315	1528.7270	0.0046	0	(14)	3.2e+002	3	K.VGGHAAEYGAEALER.M
	7545	612.2971	1833.8693	1833.8686	0.0007	0	13	1.5e+002	4	K.TYFPHFDLSHGSAQVK.G
**9**	**gi|296488813 Mass: 321384 Score: 63 Sequences: 8 emPAI: 0.01** **collagen, type VI, alpha 3-like isoform 3 [Bos taurus]**									
	**Query**	**Observed**	**Mr(expt)**	**Mr(calc)**	**Delta**	**Miss**	**Score**	**Expect**	**Rank**	**Peptide**
	73	**367.7338**	**733.4530**	**733.4487**	**0.0044**	**0**	**37**	**0.37**	**1**	**R.LVFTVR.E**

	118	374.7170	747.4194	746.4439	0.9755	0	18	60	1	K.VFAVGVR.N
	311	403.2312	804.4477	804.4494	-0.0016	0	29	5.5	1	K.ALEFVAR.N
	1605	497.7542	993.4939	993.4767	0.0172	0	22	18	2	R.DSFQEVLR.F
	1979	519.2746	1036.5346	1036.5189	0.0157	0	22	54	1	R.VAVVQYSDR.T 1974
	2977	384.2038	1149.5895	1149.5778	0.0117	1	23	17	1	R.RDSFQEVLR.F
	3391	396.8990	1187.6752	1186.6935	0.9818	2	15	78	1	R.NIFKRPLGSR.I
	7170	854.9382	1707.8619	1707.8679	-0.0060	0	63	0.0015	1	R.VAVVTYNNEVTTEIR.F
